# Nanoparticle-enabled molecular imaging diagnosis of osteoarthritis

**DOI:** 10.1016/j.mtbio.2025.101952

**Published:** 2025-06-06

**Authors:** Tianrui Zhang, Qianyi Zhang, Jingqian Wei, Quanbin Dai, Dzenita Muratovic, Wenjie Zhang, Ashish Diwan, Zi Gu

**Affiliations:** a School of Chemical Engineering, University of New South Wales, Sydney, 2052, NSW, Australia; b Jiangsu Key Laboratory of Marine Pharmaceutical Compound Screening, College of Pharmacy, Jiangsu Ocean University, Lianyungang, 222005, China; c Centre for Orthopaedic & Trauma Research, Faculty of Health and Medical Sciences, University of Adelaide, Adelaide, Australia; d School of Computer Science and Engineering, University of New South Wales, Sydney, 2052, NSW, Australia; e Spine Labs, St. George and Sutherland Clinical School, University of New South Wales, Sydney, 2217, NSW, Australia; f Adelaide Medical School, University of Adelaide, Adelaide, SA 5005, Australia

## Abstract

Osteoarthritis (OA) is the most common type of arthritis and affects patients with chronic pain, while imposing a heavy burden on public health systems worldwide. Current imaging technologies such as X-ray, MRI, and CT assist the diagnosis and monitoring of OA by providing anatomical pathological information. However, given the complex nature and progression of OA, conventional imaging technologies are limited in the molecular pathological information they are able to present and identify from the various health conditions of OA patients. Thus, nanoparticle-assisted imaging is promising to revolutionize the diagnosis and monitoring of OA, improving the sensitivity and specificity of imaging by enhancing the detection of key biomarkers such as proteoglycans, glycosaminoglycans, type II and X collagen, and inflammatory factors. In this review, the anatomical and pathological characteristics of OA, existing imaging modalities for OA diagnosis, and recent advances in the development of functionalized nanoparticles for molecular imaging of OA are summarized, highlighting the specific roles of nanoparticles in targeting biomarker molecules in different stages of OA progression. Additionally, the combined fields of artificial intelligence (AI) and imaging technology are discussed, followed by an overview of current challenges and future development of nanoparticles for molecular imaging of OA.

## Graphical abstract

**Figure:**
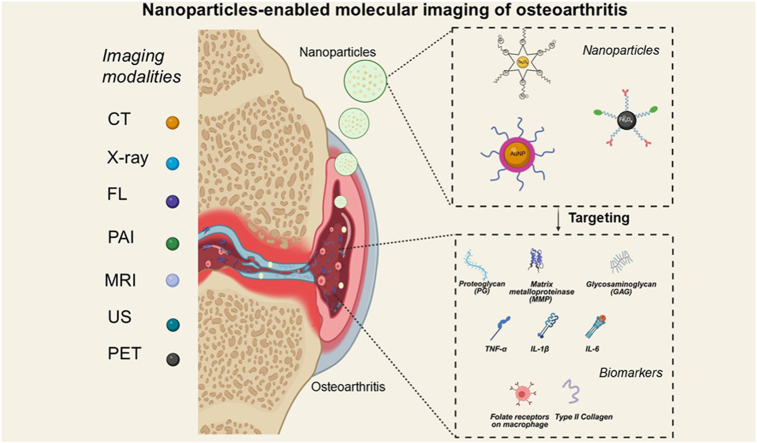

## 1 Introduction

**Fig. 1:**
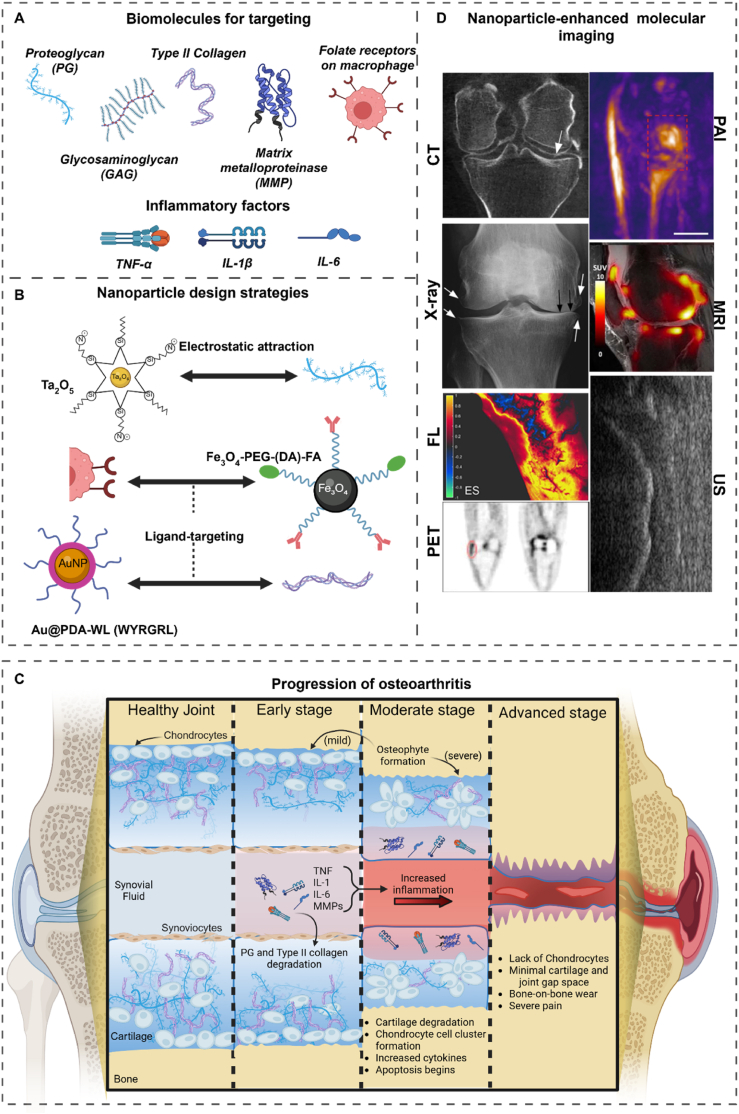
**Schematic illustration of targeted molecular imaging design and development for OA, by converging A)** biomolecules relevant to OA, **B)** imaging nanoparticle design [15,16], **C)** pathology of OA, and **D)** imaging modalities using different nanoparticles [[17], [18], [19], [20], [21], [22], [23]]. Illustrations and images from B and D are adapted from Refs. [[15], [16], [17], [18], [19], [20], [21], [22], [23]] with permission. Schematics are made with BioRender.

Osteoarthritis (OA), once considered solely a cartilage disease, is now recognized as a condition affecting the entire joint, including cartilage, bone, muscles, and ligaments. However, the most significant degenerative changes primarily occur in the osteochondral unit (cartilage-bone unit), which plays a crucial role in joint health and disease [1,2]. OA is a complex and progressive musculoskeletal disorder of the entire joint, with several risk factors identified such as elderly age, genetics, obesity, and especially any previous acute joint injury [3,4]. In 2020, 7.6 % of the global population were living with OA [5]. Currently, there is no medication approved to reverse or cure OA, with clinical care standards focused on relieving patients’ pain and symptom management. Diagnoses of OA are based mainly on patient interviews and clinical examination of symptoms, such as patient reports of serious joint pain when moving, morning stiffness phenomena, or other symptoms such as joint locking [6]. Imaging of the knee joint, such as by radiography (X-ray imaging), magnetic resonance imaging (MRI), X-ray computed tomography (CT), photo-acoustic imaging (PAI), ultrasonography (US), rescent imaging (FL) and nuclear bone scan imaging can further elucidate more complex illness conditions [7]. However, due to their limitations in sensitivity and specificity, these imaging technologies currently cannot clearly detect early-stage arthritis or differentiate conditions like rheumatoid arthritis from OA. In order to address this problem, further development is required in several related fields such as imaging technologies, understanding of OA pathology, and synthesis of targeted biomolecular nanoparticles to enable new advanced interdisciplinary therapeutic strategies for improved overall treatment. Biomolecules, such as proteoglycans (PG), glycosaminoglycans (GAG), type I, II and X collagen, and matrix metalloproteinases (MMP), are fundamental to the structure and healthy function in the bone, cartilage, and overall joint region [[8], [9], [10]]. OA progression affects the different expression levels of these biomolecules, meaning accurately measuring these changes can serve to represent various disease conditions (Fig. 1A).

Thus, the surface modification of targeting ligands and responsive motifs enables engineered nanoprobes to selectively recognize these biomolecules to improve accuracy and differentiate the contrast between normal and diseased tissues (Fig. 1B) [11]. Additionally, the recent emergence of AI technology has the potential to advance diagnostic practices with technological breakthroughs in a clinical context. Deep learning algorithms could assist in developing new tools to enhance diagnosis and prediction of future OA symptomatology and structural degradation [[12], [13], [14]].

## 2 Pathology of osteoarthritis

Historically, OA was considered generally as a bio-mechanical issue, where cartilage simply wore down over time due to daily activity, and eventually resulted in a bone-on-bone condition. Since then, advances in clinical science and biology have redefined OA as a more complex issue than just cartilage degradation. OA is now considered a complex pathology that affects the entire joint, including the synovium, ligaments, muscles, and especially the osteochondral unit (including articular cartilage and subchondral bone), as a functional and pathological entity. There are three stages: early, moderate, and severe, where not only mechanical stress, but also other pre-existing factors such as genetics, obesity, and gender play important roles in the disease [[24], [25], [26]]. The rate of the OA progression is believed to be dependent on individual’s specific physiological, metabolic and immunological factors. For instance, certain patients may be able to maintain and manage their day-to-day condition to slow progression to late-stage severe OA conditions. While in contrast, other patients may experience rapid early to severe OA, even in the absence of excessive bio-mechanical stress to their affected joints. This discrepancy has been attributed to individual variability in the pathological mechanisms of OA, such as extreme inflammation driving rapidly disease progression in certain patients [27]. Thus, a comprehensive understanding of the diverse conditions of OA and the role of associated biomolecules is essential for determining disease stage of OA upon diagnosis and for designing advanced, personalized therapeutic strategies (Fig. 1C).

### 2.1 Early osteoarthritis

The major limitation in clinical identification and diagnosis of early-stage OA is the absence of a well-defined starting point. Patients often seek medical advice only after the onset of symptoms, by which point OA may have already initiated. Therefore, the diagnosis is usually made by the physician through a face-to-face clinical assessment of the patient's symptoms. Once the diagnosis is confirmed, further evaluation should be performed using a combination of imaging techniques and grading systems to characterize disease severity on imaging findings. These may include the Kellgren-Lawrence (KL) grading system, the whole-organ magnetic resonance imaging score (WORMS), and the MRI OA knee score (MOAKS), which are discussed in detail in the following sections. In terms of pathological features, early OA is characterized by the presence of bone marrow lesions (BMLs) in the subchondral bone, mild osteophyte formation, and mild joint space narrowing. Pathological changes in the subchondral bone include increased remodelling activity, microdamage, and thinning of the subchondral plate. Concurrently, the trabecular bone exhibits increased porosity. These changes potentially reduce the mechanical buffering capacity of the bone, thereby exacerbating cartilage stress [26]. Increased porosity also enhances biochemical crosstalk between the subchondral bone and cartilage through vascular channels and remodelling zones. At this stage, although no obvious structural damage or changes in the osteochondral unit may be observed by radiographic images, patients often begin to experience symptoms such as joint pain [28,29].

Pathologically, the cartilage and bone matrix undergo significant changes, even though no major structural damage is evidenced at this early stage. Degradation of proteoglycans (especially aggrecan) and type II collagen impairs the water retention capacity of the cartilage, thereby reducing its elasticity and compressive strength [30]. Initially, transforming growth factor-beta (TGF-β), which is a cartilage protective, is overexpressed due to increased and aberrant overloading. It contributes to pathologic changes in cartilage calcification and structural changes in subchondral bone accompanied with the generation of blood vessels and nerve fibres [31]. At the same time, inflammatory cytokines (such as IL-1β and TNF-α) gradually increase in synovial fluid, stimulating the release of matrix-degrading enzymes matrix metalloproteinases (MMPs) [32], disintegrin, and metalloproteinase with thrombospondin motifs (ADAMTS-4 and ADAMTS-5), thereby further accelerating cartilage destruction [33]. The inflammatory response also leads to synovial thickening and angiogenesis, both of which contribute to aggravation of cartilage damage. During inflammatory processes, degradation products such as proteoglycans and type II collagen fragments accumulate in the cartilage matrix, signifying the transition from early to mid-stage moderate OA [34].

### 2.2 Moderate osteoarthritis

In mid-stage moderate OA, multiple adverse reactions are exacerbated due to chronic overloading, leading to joint and osteochondral degeneration, which is one of the main identifying features of this stage. Increased activity of matrix metalloproteinases (MMP-13) and aggrecanase (ADAMTS-4) accelerate the degradation of type II collagen and proteoglycans such as aggrecan, resulting in cartilage matrix loss, leading to the formation of fissures in the cartilage structure [35]. Simultaneously, cytokines secreted by polarized macrophages regulate the differentiation of mesenchymal stem cells (MSCs), thereby influencing cartilage regeneration and self-repair [36].The loss of these structural components weakens the cartilage's ability to resist mechanical stress, leaving it more susceptible to further damage [37,38]. In response, chondrocyte expression becomes more pronounced during this stage, with chondrocytes proliferating in an attempt to repair the damaged extracellular matrix, but excessive proliferation can lead to cell cluster dysfunction and accelerated cell death [39]. Apoptosis and necrosis further damage cartilage integrity and reduce repair capabilities [38].

The thickness of the subchondral plate increases, and the pronounced thickening of plate-like trabeculae continues due to increased bone formation as a response to higher mechanical loads and compensatory mechanisms attempting to stabilize the joint. Formation of BMLs occur with increased mineral density and sclerosis.

In addition, proinflammatory cytokines such as IL-6, TNF-α, and IL-1β are released in synovial fluid, which can promote OA progression by affecting normal homeostasis of the cartilage and bone tissue [40]. Studies have shown that elevated IL-6 levels are associated with pain and functional impairment in OA patients [41]. Similarly, elevated concentrations of TNF-α and IL-1β are also correlated with cartilage degradation and narrowing joint space, highlighting their potential utility as biomarkers for monitoring OA progression [42].

### 2.3 Advanced osteoarthritis

In late-stage OA, significant pathological changes occur in both cartilage and subchondral bone as the disease progresses. The most notable change is the upward movement and duplications of the tidemark, which signifies the expansion of the calcified cartilage layer accompanied by a corresponding reduction of uncalcified cartilage zone [43]. At this point, blood vessels and nerves penetrate the tidemark into the calcified cartilage layer [44]*,* potentially leading to an inflammatory response and increased pain. The detection of degradation products, such as type II collagen degradation products (e.g., CTX-II), indicates that this vascular invasion occurs alongside cartilage degradation [45,46]. Destructive changes in the osteochondral tissue also include hypo-mineralization, resulting in increased local hardness and brittleness, which in turn leads to cracks and microfractures [47].

The calcified cartilage continues to deteriorate, eventually exposing the subchondral bone to subsequent damage. Subchondral bone sclerosis, one of the main characteristics of late-stage OA, manifests as an increase in bone density. However, despite this apparent densification, the bone becomes structurally compromised and more susceptible to fragility and microdamage. Increased and abnormal subchondral bone remodelling results in formation and/or increase in BML size, which directly correlate with severity of osteochondral degeneration and increased patients’ discomfort such as pain and joint stiffness. This association is particularly pronounced when BMLs accompanied with subchondral bone cysts (SBCs) indicate serious joint damage [[48], [49], [50]]. During late-stage OA, biomolecules such as COMP and C1M serve as indicators of structural progression in osteochondral tissue by reflecting the underlying tissue metabolism and turnover and are used as valuable biomarkers for diagnosing and monitoring of disease [45,51].

From early to late stages, the progression of OA is driven by the interplay of cartilage degradation, subchondral bone remodelling, and inflammatory responses [52], with specific biomarkers reflecting the pathological changes at each stage. Monitoring these key biomarkers can provide a better understanding of OA progression and offer novel approaches for OA diagnosis and personalized treatment strategies. Next, the discussion focuses on OA imaging diagnostic approaches and developing molecular biomarkers via nanotechnology [45,50].

## 3 Clinical OA imaging and related emerging technologies

Current OA imaging modalities include conventional radiography, magnetic resonance imaging (MRI), computed tomography (CT), ultrasonography (US), nuclear medicine bone scan, and fluorescent imaging (FL). Furthermore, photoacoustic imaging (PAI), as an emerging pre-clinical technology, is already on the way to implementation for detecting other diseases in the lab and could also potentially serve as a useful strategy for OA assessment. An introduction to each imaging modality is presented along with considerations for their advantages and shortcomings (Table 1). In addition, contrast agents that enhance imaging performance are introduced (Fig. 1D) and certain nanoparticle contrast agents that are specifically designed along with the respective modalities will also be discussed comprehensively in following section. In summary, this section will highlight different approaches used to improve image quality, both from an imaging technology design perspective here and a biomolecule targeted perspective discussed later.

**Table 1:** Summary of OA-related clinical and emerging imaging modality.

Modality	Spatial Resolution	Cost	Sensitivity	Commercial contrast agent	Strengths	Shortcomings
X-ray	0.1–0.2 mm	Low	Low	Hexabrix® 320 (iodine)	Minimal radiation exposure; Quick to perform; Correlates to established KL grading system	Limited to 2D imaging; Limited to visualize soft tissue damage and cartilage degeneration; Operator-dependent; Radiation exposure
MRI	1-5 mm	High	High	Magnevist® (Gd^3+^); SPION	Non-ionizing; Excellent soft tissue and anatomical resolution for cartilage, synovium, and bone marrow lesions, several MRI-based scoring systems in clinical use	Relatively long imaging time; May be contraindicated in obese patients to fit the coil
CT	0.5-1 mm	Moderate	Moderate	Hexabrix® 320 (iodine)	High anatomical resolution of subchondral bone; Quantitative analysis in absence of contrast agent	Radiation exposure; Limited soft tissue contrast
US	50-500 μm	Low	Moderate	SonoVue®, Optison® and Definity® (microbubbles)	Non-ionizing; Quick to perform; Real-time dynamic imaging; Effective for synovial inflammation and effusion	Operator-dependent; Limited subchondral bone resolution; Inability to visualize intra-articular structures
PAI	50-500 μm	N/A	High	N/A	Non-ionizing; Enable synovial tissue oxygenation level assessment; Quantitatively assess OA disease progression	Pre-clinic modality
Nuclear Bone Scan	2-10 mm (PET)	Moderate	High	[^18^F]NaF and^18^F-FDG	Provides metabolic information on bone remodelling and inflammation; Excellent for molecular targeting; Evaluate complex pathogenesis of OA	Long imaging time; Low spatial resolution; Ionizing radiation;
FL	100-500 μm	Low	High	Indocyanine green (ICG)	Non-ionizing; Real-time guidance	Limited tissue penetration

### 3.1 Radiography

As the earliest developed technology, conventional radiographs are traditionally used as the first supplementary clinical imaging diagnostic for identifying the stages and severity of OA. Radiography generates signals by transmitting high-energy ionizing radiation through the body, where different tissues absorb the X-rays to varying degree depending on their density and atomic composition [53]. Dense structures such as bones absorb more radiation and appear brighter on the resulting image, making this technique particularly effective for visualizing bones, fractures, and calcifications, but offers limited contrast for soft tissues unless contrast agents are used. Nanomaterials with a high K-edge, which typically means the element has a high atomic number (Z), are normally used for X-ray contrast agents. They provide strong attenuation and absorption of high X-ray energies compared to elements in biological tissues like carbon, oxygen, nitrogen, and hydrogen [54]. While conventional radiography remains the traditional imaging modality of OA, there is still a clear need to address its notable limitations such as low sensitivity and limited clinical correlation [55].

The Kellgren-Lawrence (KL) grading system was designed to assess the severity level of knee OA under clinical assessment of radiographic indications [56], with classified radiological features assigned to different stages of OA (Table 2) [57]. This grading system mainly relies on individual skill and experience [58], where some radiologists consider KL grades <2 to represent no OA symptoms [59] while others considered KL grades <2 as an early stage of OA [60]. This high level of subjectivity can lead to a wide divergence in patient experiences. The KL grading system highlights the difficulties that conventional radiography encounter when imaging and diagnosing soft tissue joints.

**Table 2:** Kellgren-Lawrence (KL) grading for OA [57].

Grades	Description
0 (none)	No X-ray changes of OA
1 (doubtful)	Doubtful joint space decreasing and possible osteophytic lipping
2 (minimal)	Determinate osteophytes and possible joint space narrowing
3 (moderate)	Moderate multiple osteophytes form, determinate joint space narrowing, some sclerosis and possible deformity of bone ends
4 (severe)	Large osteophytes form, remarkable joint space narrowing is shown, severe sclerosis and determinate deformity of bone ends

Furthermore, Amin et al. analyzed radiography versus MRI data from 317 participants, in which 87 % of them underwent both follow-up knee MRI and radiography at either 15 months, 30 months, or both. It demonstrated the inherent limitations on sensitivity towards capturing soft tissue damage and cartilage degeneration in radiography, whereas MRI was able to accurately represent cartilage loss in certain knee joint areas such as the posterior femur [61]. Therefore, MRI is increasingly used to evaluate joint health in individuals with an early symptomatic onset of OA, as it can detect changes in cartilage composition, including reactive bone marrow lesions, soft tissue inflammation, and cartilage degeneration during the initiation and progression of OA.

### 3.2 Magnetic resonance imaging

MRI serves as a complementary or a standalone imaging analysis to achieve a more comprehensive diagnosis due to better detection of soft tissue sensitivity and early cartilage degeneration [55]. It is a non-invasive imaging technique that uses strong magnetic fields and radiofrequency pulses to provide prominent contrast between soft tissue and bone enhancing the ability to detect early‐stage OA-related changes. [62]. When an external magnetic field aligns the spins of water protons in the body, a brief radiofrequency pulse is then applied and temporarily disturbs this alignment. As the protons return to equilibrium, radiofrequency signals are emitted and received by coils to generate images. For example, T_1_-weighted MRI often limits the detection of articular cartilage defects and meniscal abnormalities due to insufficient contrast between the cartilage surface and synovial fluid. Whereas, T_2_-weighted MRI provides good contrast between the cartilage surface and joint effusion to examine focal areas of delamination or other defects [63]. However, the application of T_2_-weighted MRI sacrifices the internal cartilage signal due to partial components in cartilage having short T_2_ [64].

The Whole-Organ Magnetic Resonance Imaging Score (WORMS) and the MRI OA Knee Score (MOAKS) are comprehensive MRI-based scoring systems designed for detailed assessment of knee OA. WORMS was developed in 2003 as one of the first systems for whole-organ assessment. It provides a comprehensive evaluation of all joint tissues affected by OA, including cartilage, menisci, bone marrow, osteophytes, synovitis, effusions, and the integrity of ligaments and tendons [65]*.* Building upon WORMS and introduced in 2011, MOAKS incorporates updates and refinements such as more detailed cartilage assessments, improved detection of bone marrow lesions, and the inclusion of additional features like infrapatellar fat pad changes. These updates enhance MOAKS' ability to capture OA's progressive nature and provide a more nuanced understanding of the disease. Both systems are pivotal in advancing OA research, facilitating the monitoring of disease, progression, and improving clinical management of this debilitating condition [66].

Detecting joint cartilage degeneration in the early stages of OA can help patients start treatment earlier to delay or even avoid total joint replacement surgery [67]. Clinically, MRI obtains the cartilage change information by detecting fast spin echo (FSE) sequence, gradient echo sequence, inversion recovery sequence, etc. However, the accuracy and sensitivity are not high enough, thus necessitating the introduction of an external material as a contrast agent.

Cartilage degeneration in OA is often characterized by loss of extracellular proteoglycans that contain negatively charged sulfate and carboxylate groups, constituting a fixed charge density [68]. Sodium (Na^+^), the majority of positively charged ions in synovial fluid and cartilage, distributes into cartilage in proportion to this fixed charge density that can be measured by MRI to determine the proteoglycans charge density [69]. The compositional MRI assessment associated with sodium (Na^+^) and gadolinium-based contrast agents are applied to focus on the molecular status [70]. Delayed gadolinium-enhanced MRI of cartilage (dGEMRIC) is a technology developed to map cartilage degeneration, utilizing an anionic paramagnetic contrast agent gadolinium diethylenetriaminepentaacetic acid (Gd-DTPA^2−^) that distributes in the cartilage in an inverse relationship to the GAG content [71]. The concentration of Gd-DTPA^2−^ can be calculated from pre- and post-contrast T_1_ values since more Gd-DTPA^2−^ is distributed within the cartilage matrix in conditions of GAG loss in early OA. However, the molecular weight of Gd-DTPA^2−^ is low, leading to the quick elimination and short residence time in vivo [72]. Paramagnetic nanoparticles composed of unpaired electrons can enhance T_1_-weighted MRI by accelerating the longitudinal relaxation of nearby hydrogen protons [73]. This interaction with surrounding water protons increases the efficiency of energy transfer during the relaxation process, resulting in a reduction of T_1_ relaxation time. Consequently, regions containing paramagnetic agents exhibit increased signal intensity and appear brighter on T_1_-weighted images, improving the contrast between different types of structure and tissues. Ouyang et al. further developed advanced MRI capabilities for OA diagnosis by synthesizing a gadolinium-based contrast agent (GdPDW), showing an obvious concentration-dependent brightening effect under T_1_-weighted MRI [74]. The longitudinal relaxation rate (r_1_) was measured to be 8.29 mM^−1^s^−1^, which is higher than the value (2.64 mM^−1^s^−1^) of clinical commonly used T_1_ contrast agent Magnevist® (Gd^3+^-DTPA). Data from in vivo studies of an OA mice model showed significant T_1_-MRI signal intensity remained after 12 h post-injection in the GdPDW-treated OA knees, compared with the PBS-treated mice. However, gadolinium contrast agents can pose a nephrotoxicity risk to humans, and the toxicity of Gd^3+^ released from the complex causes severe fibrotic disorder to people with acute kidney injury [75]. In that case, the careful assessment of kidney function is necessary before administration of gadolinium contrast agent, and alternative contrast agents or imaging modalities should be considered in patients with severe kidney disease.

Thus, there is increasing interest in developing gadolinium-free contrast agents. Superparamagnetic nanomaterials enhance T_2_-weighted MRI by inducing local magnetic field inhomogeneities that accelerate the transverse relaxation of surrounding hydrogen protons [73]. Panahifar et al. demonstrated a superparamagnetic iron oxide nanoparticle functionalized with bisphosphonates (SPIONs-ALN), with an approximate size of 17 nm, for diagnosis and monitoring of bone disorders with MRI [76]. Generally, SPIONs are used as T_2_ contrast agents unless the size decreased, upon which the T_2_-weighted imaging activity will be weakened together with the increase of T_1_-weighted activity [77].

### 3.3 Computed tomography

Computed tomography (CT) uses a combination of X-rays from multiple angles to pass through the body and differentiate tissue density via a detector to measure the intensity of transmitted X-rays, which are attenuated to varying degrees when passing through different tissues [23]. It has high spatial resolution and superior detection of subtle changes in subchondral bone, such as overall bone tissue mineralization, microtrabecular structure and size of osteophytes in OA. The underlying principle of using high K-edge nanomaterials in CT is similar to that in general X-ray imaging [78]. In both modalities, contrast is generated based on the differential attenuation of X-ray photons as they pass through materials of varying density and atomic composition. High atomic number elements exhibit greater photoelectric absorption, particularly near their K-edge, resulting in enhanced contrast [79].

However, due to radiation exposure and high cost, CT is often used for complex cases where MRI or radiography cannot meet diagnostic demand, such as identifying microfractures or cystic changes in the subchondral bone [80]. In combination with a contrast agents, CT has an increased potential to visualize and quantify soft tissue composition and properties. Currently available CT contrast agents are small anionic molecules that are repelled by the negatively charged cartilage, which hinders intra-tissue penetration and shortens intra-tissue retention time [81]. Anionic ioxaglate (Hexabrix® 320), an iodine-based CT contrast agent, is usually introduced to further improve the accuracy of detection between cartilage and surrounding tissues, but it is limited in differentiating between components having similar x-ray attenuation and rapid clearance from the joint [82].

Recently, Jäntti et al. [15] reported on use of cationic tantalum oxide nanoparticles (Ta_2_O_5_-cNPs) as μ-CT contrast agents for cartilage, providing a quantitative evaluation between the diffusion time *τ* and cartilage structure. Furthermore, a correlation between Ta_2_O_5_-cNPs partition, superficial PGs, and collagen content could be observed after 6 h, indicating sufficient contrast agent concentration accumulated in the superficial cartilage zone to produce signals for OA detection. Other efforts to improve CT imaging published by Chatzaki et al. [83] investigated gold nanoparticles coated by hyaluronic acid (HA-AuNPs), which lead to a 7.1-fold CT signal enhancement after 2-h of reaction once switched on. It is due to the increase of reactive oxygen species in cartilage degeneration that triggers the breakdown of the HA coating, activating the contrast agent’s CT signal to monitor OA progression or detect inflammation.

### 3.4 Ultrasonography

Ultrasonography (US) is a non-invasive diagnostic technology that uses high-frequency sound waves emitted through the body to visualize real-time internal body structures. As these sound waves encounter tissue and interfaces they are partially reflected to a transducer, which processes signals to reconstruct images, especially effective for soft tissues and joint structures [84]. Unlike radiography, US can detect synovitis, effusion, osteophytes and cartilage morphological changes, providing additional diagnostic information, with a study finding remarkable sensitivities of 97 % for detecting synovitis and joint effusion [21]. The advantage over MRI lies in the ability of US to demonstrate similar inflammatory OA features at a lower cost, with non-invasive and dynamic visualization with patients in weight-bearing position. While US is cost-effective and widely available in clinical settings for quick knee imaging assessment, it is restricted by the inability to visualize intra-articular structures and the poor contrast caused by fat and air [85]. US waves cannot penetrate into bone, which makes it difficult to accurately identify some early key pathological changes in subchondral bone marrow lesions [86]. The introduction of contrast agents such as FDA-approved SonoVue®, Optison®, and Definity® improves the effect and accuracy of US, based on the acoustic response of microbubbles or nanoparticles under ultrasound [87]. These particles generate harmonic vibrations under ultrasound excitation, scatter sound waves, and enhance imaging contrast, significantly improving the resolution of tissues [88]. In recent years, the application of auxiliary technologies such as contrast-enhanced ultrasound (CEUS) has further enhanced the diagnostic ability of ultrasonography for OA. CEUS uses microbubble contrast agent SonoVue® to improve image contrast, especially in the evaluation of synovitis, and can quantitatively analyze lesion microcirculation and hemodynamic [89].

### 3.5 Photoacoustic imaging

Photoacoustic imaging (PAI), an emerging non-ionizing and non-invasive imaging technique, is a new promising modality for diagnosis and monitoring of OA progression, with success in small animal study [90]. PAI delivers pulsed laser directly to biological tissue, which induces a rapid, localized temperature increase resulting in a thermoelastic expansion that generates ultrasonic waves [91]. These ultrasound waves propagate through the tissue and can be detected and traced back to their point of origin using time-of-flight measurements, enabling precise spatial localization and image display. PAI provides higher-resolution small finger joint imaging and functional evaluation capabilities by converting optical signals into acoustic signals [92]. For example, it has been used to non-invasively assess synovial tissue oxygenation levels in OA mice. Longitudinal monitoring of synovial tissue using PA imaging showed increased vascularity and decreased oxygen saturation, which correlated with histological grades of cartilage damage [93]. On the other hand, multispectral quantitative photoacoustic tomography (qPAT) has been used to image hemoglobin concentration and oxygen saturation in finger joints, revealing increased angiogenesis and hypoxia in OA joints, which are considered to be indicators of OA disease progression [94].

PAI also uses cationic probes that specifically bind to anionic glycosaminoglycans to enhance signal intensity, bringing new possibilities for early diagnosis and accurate evaluation of OA [95]. Gold nanoparticles (AuNPs) are particularly effective for PAI due to their strong optical absorption in the near-infrared (NIR) region, which corresponds to the biological tissue transparency window [96]. Upon pulsed laser excitation, they rapidly convert absorbed light into heat, generating thermoelastic expansion and consequently a strong photoacoustic signal. The localized surface plasmon resonance (LSPR) of AuNPs enhances this photothermal effect, making them excellent candidates for high-contrast, high-resolution PAI. Due to the large specific surface area, low toxicity, and especially high thermal conversion efficiency, AuNPs are widely used in PAI [97]. In a study using an Au@PDA-WL nanoprobe, researchers demonstrated the ability to monitor early cartilage degeneration by targeting collagen II peptides, which are overexpressed in OA cartilage.

Despite the great potential for preclinical research of PAI, studies have been conducted ranging from small animal imaging to pilot studies in humans [92]. It has been reported that a multispectral quantitative PA tomography was employed to investigate distal interphalangeal joints of OA patients, displaying significantly elevated water content, decreased hyperbaric oxygen, and increased acoustic velocity compared with normal joints [92].

### 3.6 Nuclear bone scan imaging

Nuclear medicine bone scan, known as bone scintigraphy, is used by physicians to differentiate bone conditions that also present with joint pain or skeletal abnormalities, such as OA, inflammation, osteomyelitis, neoplastic, and traumatic bone pathologies by analyzing the pattern, intensity, and location of tracer uptake [98]. The technique involves the injection of a radiopharmaceutical (typically technetium-99m-labeled diphosphonates) [99], that selectively bind to hydroxyapatite crystals in bone, which accumulates in areas where high bone turnover and inflammation is occurring [100]. It offers the unique ability to provide quantitative information about molecular and metabolic activities, which is necessary for evaluating the complex pathogenesis of OA. ^18^F-Sodium Fluoride ([^18^F]NaF), taken up in areas of newly mineralized bone and correlated with bone histomorphometry, has been the primary positron emission tomography (PET) tracer applied for OA study due to its unique capacity to interrogate bone remodelling [22]. It is reported that subchondral bone metabolic activity was significantly increased in regions of normal-appearing bone and adjacent cartilage in an OA-group compared with healthy group [22]. Paired with MRI, [^18^F]NaF served as a marker of bone metabolism and dynamic contrast-enhanced (DCE)-MRI of synovial inflammation, providing direct in vivo evidence of associations between synovitis and adjacent metabolic bone activity and osteophyte formations [101]. The other radioactive tracer ^18^F-fluorodeoxyglucose (FDG) also indicates the presence of inflammation suggestive of synovitis and correlates with OA severity [20], but the lack of high anatomical resolution needs to be overcome by PET/CT and PET/MRI hybrid imaging [102].

### 3.7 Fluorescence imaging

Fluorescence (FL) imaging is a non-ionizing, rapid, and cost-effective technique that offers high sensitivity for detecting specific biomolecular targets and enables real-time visualization of biological processes at both cellular and tissue levels using fluorescent dyes [103]. Fluorescent dyes emit specific fluorescent signals under particular excitation by light irradiation, in which unique signals are collected by digital devices, and produce images that reflect the spatial and quantitative distribution of disease activity [104]. The current FDA approved clinical NIR-I dye indocyanine green (ICG), assisted with surgical decision-making, is employed to perfuse in soft tissue, creating opportunities for early and minimally-invasive assessment of tissue vascularity in joints that may cause post-traumatic arthritis [23]. A pilot study showed that ICG signal was within the microvasculature of a representative knee joint, demonstrating the potential for visualization of small calibre vessels in patient. The traditional FL imaging modality of the NIR first window (NIR-I, 400–900 nm) is more damaging to tissue due to the harmful photon scattering [105]. The limited penetration depth of short-wavelength FL, high injection dose, and in vivo tissue autofluorescence in NIR-I window result in restricted FL depth resolution for bioimaging. Therefore, FL imaging in the second NIR window (NIR-II, 1000–1700 nm) has become a promising modality for bioimaging, with the NIR-II region being virtually harmless to tissue due to its longer wavelengths and lower frequencies, allowing visualization of deeper tissues [106]. Some NIR-II fluorescent dye labeled nanoprobes paired with hybrid imaging modalities will be discussed in the following section.

## 4 Biomarker molecule-targeted nanoparticles-assisted OA imaging

As discussed above, conventional imaging modalities used for OA diagnosis are limited by low tissue contrast, high radiation dose, insufficient imaging resolution, or limited detection of internal structures, which can compromise the diagnostic accuracy and pose health risks to patients. Moreover, the existing diagnostic imaging modalities are limited by providing only anatomical structure but not pathological information at the molecular level. Therefore, the development of various functional nanoprobes to address these challenges and remedy the limitations of the traditional diagnostic imaging would not only improve imaging technology, but also provide a critical measurement of OA progression on a molecular level [107,108]. Thus, emerging imaging techniques and development of nanoparticle-derived imaging contrast agents are required to address concerns regarding efficacy, urgently needed resolution enhancement, and limited retention period [109]. Several key biomolecules have been identified for discussion in the following section, due to their key roles in OA and joint cartilage health, and the ongoing efforts to design biomolecule nanoparticle imaging agents specifically targeting them (Table 3). Further considerations are also given to the unique considerations these biomolecules may also require with regards to their combined suitability towards various imaging technologies covered earlier.

**Table 3:** Nanoparticle-based contrast agents for biomarker-targeted imaging in OA.

Nanoparticles	Receptor (Target)	Targeting mechanism	Animal model	Imaging modalities	Imaging performance	Ref
SPIO@PEG nanoparticles conjugated with peptide ligand WYRGRL	α1 chain (COL2A1)	Ligand-receptor binding	Papain-induced OA model in New Zealand white rabbits	MRI	Dual-mode T_1_–T_2_ MRI; enhanced cartilage retention; improved lesion differentiation using logical AND map	[11]
NIF-MabCII nanoparticles (monoclonal antibodies MabCII with NIF dyes)	Type II collagen	Antibody-antigen interaction	Post-traumatic OA model induced by compressive loading in C57BL/6 mice	IVIS	IVIS-based detection of early cartilage damage; selective accumulation of targeted nanosomes in damaged cartilage; signal correlated with histopathology	[111]
Au@PDA-WL nanoparticles conjugated with peptide ligand WYRGRL	α1 chain (COL2A1)	Ligand-receptor binding	ACL + MM transection-induced OA model in BALB/C female mice (7–8 weeks old)	PAI	Strong PAI signal enhancement in healthy cartilage; collagen II-targeted retention; reduced signal in degenerated cartilage; signal correlated with matrix integrity	[95]
CMFn@HCQ nanoparticles (ferritin nanocages, Cy5.5 dye, quencher)	MMP-13	Enzyme-responsive activation	Papain-induced OA model in C57BL/6J mice	Fluorescence imaging	MMP-13 responsive “turn-on” NIR fluorescence; signal correlated with OA severity; prolonged joint retention (≥14 days); cartilage-targeting via collagen II peptide	[113]
Probe 2 nanoparticles (peptide substrate, Cy5.5 dye, BHQ-3 quencher)	MMP-13	Enzyme-responsive activation	Sprague–Dawley rats (250–300 g); OA induced by ligament transection and total meniscectomy; imaging performed 8 weeks after surgery.	Fluorescence imaging	Probe 2 enabled in vivo detection of MMP-13 activity using fluorescence tomography. OA cartilage showed 3.8 × higher signal than normal. Signal reduced after MMP-13 inhibitor treatment.	[115]
MMPSense680 nanoparticles	MMPs	Enzyme-responsive activation	Surgical destabilization of the medial meniscus (DMM) in male CD1 mice	Fluorescence imaging	Serial NIR imaging of MMP activity; fluorescence in DMM knee increased steadily over 8 weeks; signal correlated with histological cartilage damage and chondrocyte death; early detection from week 1; signal localized to joint	[116]
ERMs@siM13 nanoparticles (MMP13-cleavable PEG shell, Cy5 fluorophore, BHQ-3, cRGD ligands, siM13)	MMP-13	Enzyme-responsive activation and integrin binding	Destabilization of the medial meniscus (DMM)-induced post-traumatic OA model in C57BL/6 mice	Fluorescence imaging	Cy5 fluorescence-based detection of cartilage MMP13 activity; signal increased with disease severity and reduced after therapy; allowed non-invasive monitoring of therapeutic efficacy	[117]
CA^4+^, gadoteridol, BiNPs	Proteoglycans	Electrostatic attraction	Ex vivo bovine cartilage plugs (n = 27) from mature patellae; divided into three groups: intact, proteoglycan-depleted (via trypsin digestion), and mechanically injured (via 500 g drop impact)	CT	QDECT enabled simultaneous PG and water mapping (CA^4^^+^/gadoteridol); BiNPs enhanced surface contrast for lesion detection; improved early-stage diagnostic sensitivity at 2 h	[119]
Hexabrix™ nanoparticles	Proteoglycans	Electrostatic repulsion	Rat knee joints (femur, tibia, patella); ex vivo cartilage assessment using contrast equilibration protocol	EPIC-μCT	MicroCT with anionic contrast agent (Hexabrix) enabled 3D quantification of cartilage volume, thickness, and X-ray attenuation (related to PG content); non-destructive, high-resolution alternative to histology	[120]
Ta_2_O_5_-cNPs nanoparticles	Proteoglycans	Electrostatic attraction	Ex vivo osteochondral samples from healthy equine stifle joints (medial femoral condyle and distal intertrochlear groove)	μCT	μCT detected depth-wise Ta_2_O_5_-cNP diffusion; strong correlation with PG content and cartilage stiffness; high sensitivity in superficial zone	[15]
^99m^Tc-NTP 15-5 nanoparticles	Proteoglycans	Electrostatic attraction	Destabilization of the medial meniscus (DMM) surgically induced in male C57BL/6 mice; intra-articular sprifermin (1 or 10 μg) or PBS administered post-surgery	Radiotracer imaging	SPECT-CT visualized tracer uptake within 30 min; ^99m^Tc-NTP 15-5 specifically accumulated in cartilage; uptake reflected disease progression and increased with sprifermin treatment	[121]
Ta_2_O_5_ nanoparticles (cationic and neutral)	Glycosaminoglycans (GAGs)	Electrostatic attraction	Ex vivo human metacarpal phalangeal joints from cadaveric donors were used to assess cartilage GAG content and biomechanic	CT	CECT with cationic Ta_2_O_5_ nanoparticles (NP1) showed strong correlation with cartilage GAG content (R^2^ = 0.90) and equilibrium modulus (R^2^ = 0.83); neutral NP2 correlated with stiffness and porosity. Enabled layer-specific imaging and differentiation of OA severity	[122]
PLL-MNPs nanoparticles	Glycosaminoglycans (GAGs)	Electrostatic attraction	Destabilization of the medial meniscus (DMM) in Kunming mice; contralateral knees used as controls.	PAI	Photoacoustic imaging (PAI) with poly-L-lysine melanin nanoparticles (PLL-MNPs) enabled real-time, non-invasive detection of glycosaminoglycan (GAG) loss. PA signal intensity declined progressively over 10 weeks, correlating well with histology and preceding X-ray-detectable changes	[123]
CPC–IOX nanoparticles	Glycosaminoglycans (GAGs)	Electrostatic attraction	Intra-articular injection in healthy rat knee joints; ex vivo tibial joint imaging	CT	CECT enabled clear delineation of cartilage from bone and soft tissue using only 0.5–1 mg I/mL of cationic CPC–IOX; signal intensity strongly correlated with GAG content (R^2^ = 0.91), due to enhanced intra-cartilage penetration via electrostatic attraction to negatively charged GAGs	[81]
Fe_3_O_4_-PEG-(DA)-FA nanoparticles	Folate receptors (FRs)	FR-mediated endocytosis	Inflammatory arthritis model in BALB/c mice with intra-articular inflammation in both hind legs	MRI	Dual-mode T_1_/T_2_-weighted MRI enabled by light-triggered aggregation of Fe_3_O_4_-PEG-(DA)-FA nanoparticles; signal switch from T_1_ to T_2_ achieved via laser-induced clustering (r_2_/r_1_ increases from 2.36 to 19.63); enhanced retention in inflamed tissue due to in situ aggregation prevents re-entry into circulation; clear signal enhancement and sustained contrast observed in vivo	[16]
Q-Dex nanoparticles	C-type lectin receptors (DC-SIGN, L-SIGN)	Receptor-mediated endocytosis	Obese C57BL/6 mice with macrophage-rich visceral adipose tissue induced by high-fat diet.	Fluorescence imaging	High-resolution NIR fluorescence imaging of macrophages; enhanced signal quantification and photostability across in vivo, ex vivo, and in situ modalities.	[130]

### 4.1 Type II collagen

Type II collagen is a major component, comprising 90–95% of the total collagen content, of articular cartilage that supports the tensile strength and structural integrity of cartilage. Under normal conditions, type II collagen maintains a dynamic equilibrium during its synthesis and degradation processes, which can be disrupted by OA progression, leading to the gradual degradation of cartilage structure caused by the disruption of equilibrium. In OA joint, the amount of type II collagen degradation increases significantly, which is mediated by the overactivity of collagenase. A series of degradation fragments is formed by cleavage of type II collagen, including the C2C epitope (COL2−3/4Clong mono) and the C1,2C epitope (COL2−3/4Cshort). The C2C epitope is a specific marker of type II collagen degradation, reflecting the degradation level of type II collagen in joint cartilage, whereas the C1,2C epitope has lower specificity, detecting degradation fragments of both type I and II collagens [110]. By measuring the ratio of these degradation products to the CPII synthesis marker, the metabolic balance of cartilage and the progression of OA can be assessed [110].

**Fig. 2:**
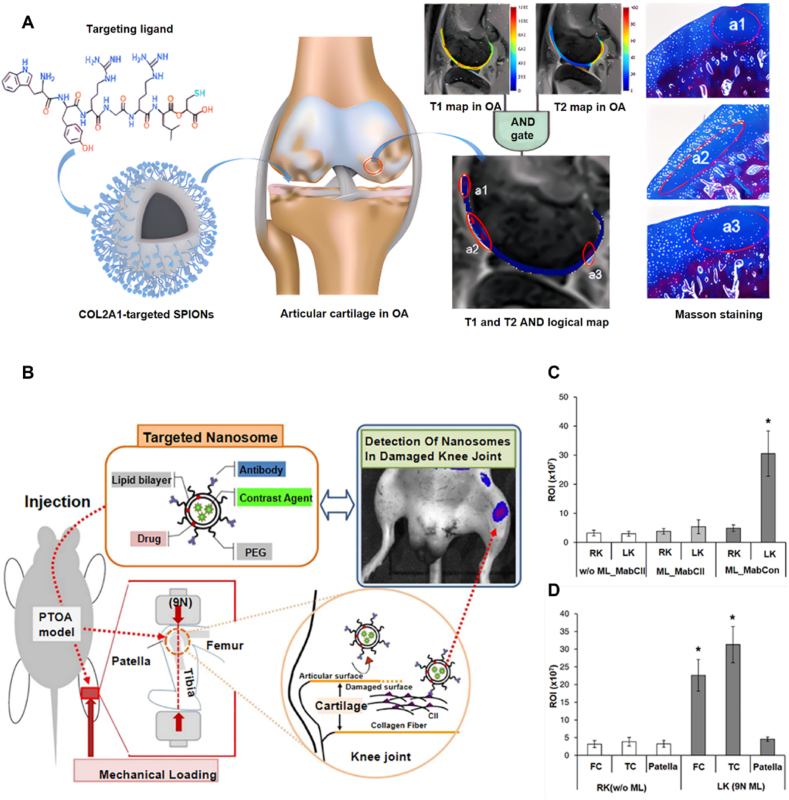
**Imaging nanoparticle targeting type II collagen. A**) Mechanism of modified COL2A1-target SPIONs nanoparticle synthesis and targeting to cartilage [11]. **B)** Schematic representation of antibody-targeted nanoparticles binding to damaged cartilage in a post-traumatic OA (PTOA) mouse model [111]. **C)**. Fluorescence intensity in loaded knees [111]. **D)** Fluorescence intensity of dissected femoral condylar cartilages (FC), tibial condylar cartilages (TC), and patellae of the mice [111]. Adapted from Refs. [11,111] with permission.

The degradation of type II collagen in the early stage of OA enables the precise detection of early cartilage degeneration. Many nanoparticles have been developed as contrast agents to target type II collagen and improve the resolution and tissue contrast of OA imaging. For example, Wu et al. [11] developed an ultra-small superparamagnetic iron oxide nanoparticle (SPIO@PEG) smaller than 6 nm to penetrate the extracellular matrix of cartilage with good retention, enabling high sensitivity detection of early micro pathologies (Fig. 2A). The cartilage-targeting peptide WYRGRL which targets the α1 chain of type II collagen existing in cartilage ECM conjugated on the SPIO nanoparticle can combine to the α1 chain (COL2A1) of type II collagen, thus making SPIO@PEG nanoprobes accumulate at the area where type II collagen is concentrated. The MRI results showed that T_1_ and T_2_ relaxation time of the SPIO@PEG injected cartilage decreased obviously compared to the cartilage without nanoprobe injection. Anti-type II collagen antibody is another targeting ligand with specific recognition of type II collagen. While this study demonstrated strong imaging efficacy of ultra-small SPIO@PEG nanoparticles, it did not assess their toxicity or biocompatibility in vivo. Considering reports of SPION-related oxidative stress and long-term retention in other studies, additional safety evaluations are warranted for future clinical application.

Nanoparticles targeting type II collagen are also used as PAI contrast agent to improve the tissue signal contrast. For example, Shen et al. [95] developed a nanoprobe using a same peptide ligand WYRGRL modified on polydopamine loaded gold nanoparticles (Au@PDA-WL NPs), targeting type II collagen by the combination of α1 chain (COL2A1) of type II collagen and WYRGRL. The results indicated that the signal intensity in mice joint with Au@PDA-WL injection increased significantly after 30 min compared to the non-injected mice joint. In addition, cytotoxicity assays in ATDC5 cells demonstrated that Au@PDA-WL NPs exhibited low toxicity (<30 % inhibition) even at high concentrations (up to 360 pM), and the presence of polydopamine coating further reduced cytotoxicity compared to uncoated AuNPs, confirming their good biocompatibility. Although short-term clearance was observed within 24 h post-injection, long-term in vivo retention and potential accumulation of AuNPs were not assessed in this study.

Cho et al. [111] developed a contrast agent (NIF-MabCII) with a dimension of 100–200 nm in diameter by combining monoclonal antibodies against type II collagen (MabCII) with near-infrared fluorescent (NIF) dyes (Fig. 2B). NIF-MabCII nanoparticles target type II collagen by the agglutination effect between type II collagen and MabCII. In a compressive-loading induced murine PTOA model, IVIS imaging demonstrated selective accumulation of NIF-MabCII at the damaged cartilage site, with no detectable signal in healthy contralateral joints or in mice injected with non-targeted controls. Fluorescence signals in loaded knees reached a radiant efficiency of over 14 × 10^7^ photons/s/cm^2^/sr (μW/cm^2^), indicating robust probe accumulation (Fig. 2C). Further confirmation of the dissected joints revealed that fluorescence was limited to the femoral and tibial articular cartilages, with negligible signal in the patellar cartilage or surrounding tissues (Fig. 2D). This antibody-conjugated liposome system improves detection and intervention in early OA lesions by precisely targeting type II collagen in cartilage.

### 4.2 Matrix metalloproteinases

Matrix metalloproteinases (MMPs) serve as key enzymes within the cartilage matrix responsible for remodelling and depredating of ECM components. MMPs are zinc-dependent endopeptidases, with specific members such as MMP-1, MMP-3, and MMP-13 highly expressed in osteoarthritic cartilage. Typically, MMP activity is tightly regulated in normal physiological conditions, but completely reverses in OA. Pro-inflammatory cytokines such as interleukin-1 (IL-1) and tumour necrosis factor-α (TNF-α) induce excessive MMP expression and activation. The resultant dysregulated MMP activity leads to excessive ECM degradation, thereby triggering and exacerbating cartilage deterioration. Consequently, MMPs not only contribute to the degradation and weakening of cartilage matrix but also play an important role in subchondral bone changes. These changes include bone resorption and sclerosis, which are hallmark features of OA progression, making them valuable biomarkers for assessing severity and progression [112].

**Fig. 3:**
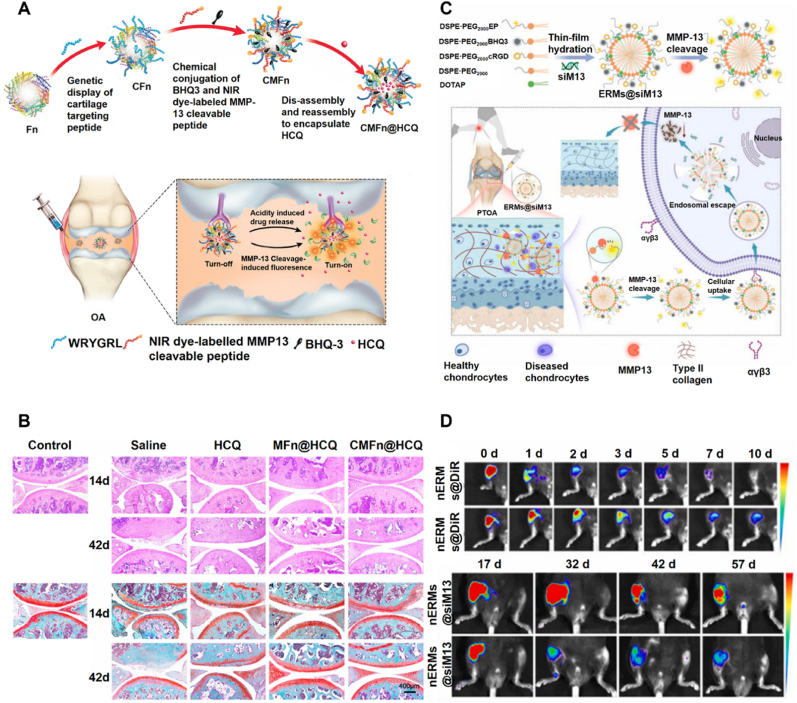
**Imaging nanoparticles targeting MMPs. A)** Schematic graph of the nanoparticle CMFn@HCQ which targets to cartilage and working as a MMP-13/pH dual-stimuli activatable theranostic nanoprobe for MMP-13 imaging and precision therapy of OA [113]. **B)** Cartilage sections with different dyes (safranin-O (top) fast green (bottom)) after treatment for 14 and 42 days. Scale bar: 400 μm [113]. **C)** Mechanism of nanoparticle ERMs@siM13 enhance imaging in the early-stage posttraumatic OA (PTOA) mouse model [117]. **D)** Representative fluorescence images of PTOA mouse joints were taken at various time points after ERMs@DiR and nERMs@DiR injections, and ERMs@siM13 and nERMs@siM13 were administered every 5 days for 9 doses, with imaging tracking their distribution and effects [117]. Adapted from Refs. [113,117] with permission.

Many nanoparticles that target MMP, especially MMP-13, have been developed as contrast agents to improve the resolution and tissue contrast. For example, Chen et al. [113] developed a novel dual-stimuli-responsive theragnostic nanoprobe (CMFn@HCQ) based on genetically engineered ferritin nanocages, approximately 20 nm in diameter, and self-assembled from 24 subunits (Fig. 3A). The surface of the nanocages was modified with cartilage-targeting peptide (WYRGRL) and a chemically conjugated MMP-13 cleavable peptide substrate tagged with a near-infrared dye (Cy5.5) and a quencher (BHQ3) to enable MMP-13-responsive fluorescence activation. A crucial step in the pathogenesis of OA is the elevated expression of MMP-13, which contributes to cartilage ECM breakdown. This degradation process can lower the joint pH to around 6.0, resulting in an acidic microenvironment in OA cartilage [114]. The internal nanocage cavity was loaded with the anti-inflammatory drug hydroxychloroquine (HCQ), enabling pH-responsive drug release under the acidic OA microenvironment. In the OA joint, which is characterized by overexpressed MMP-13 and acidic pH, the nanoprobe activated through MMP-13 cleavage of its substrate, resulting in fluorescence activation for real-time imaging. Simultaneously, the acidic conditions triggered nanocage disassembly, enabling the sustained release of HCQ, thereby showing significant alleviation of OA progression in vivo (Fig. 3B). Biocompatibility was confirmed by in vitro CCK-8 and hemolysis assays, showing no cytotoxicity of CMFn and enhanced cell viability at 4 μg/mL HCQ. In vivo, no adverse effects were observed, consistent with previous studies using MMP-13-targeted imaging probes. This multifunctional nanoprobe, with precise targeting, dual-stimuli responsiveness, and excellent biocompatibility, presents a promising platform for imaging-guided therapy of OA with strong potential for clinical applications. Elsewhere, Ryu et al. [115] developed and optimized a nanostructured fluorogenic probe targeting MMP-13 for sensitive and specific detection of OA. These nanoparticles were designed by conjugating a near-infrared fluorescent dye (Cy5.5) and a quencher (BHQ-3) to a peptide substrate, forming nanoparticles that remain quenched under normal conditions but emit fluorescence upon cleavage by MMP-13. The optimized nanoparticle named Probe 2 showed excellent specificity for MMP-13 compared to other proteases such as MMP-2 and MMP-9, with in vitro studies showing a 36-fold increase in signal intensity at 15 nM of MMP-13. In an OA-induced rat model, these Probe 2 nanoparticles provided 3.8 times higher fluorescence intensity in OA cartilage compared to normal cartilage, with the signal significantly reduced to baseline levels when MMP-13 inhibitors were applied. The study highlights the potential of these nanostructured probes for precise OA imaging and therapeutic evaluation, leveraging their nanoscale architecture for enhanced targeting specificity and efficient detection.

In another work of improving nanoprobe imaging precision, Leahy et al. [116] used a matrix metalloproteinase (MMP)-sensitive near-infrared fluorescence (NIRF) nanoparticle probe, MMPSense680, to monitor OA progression by detecting MMP activity both in vitro and in vivo. This nanoparticle-based probe exhibited enhanced fluorescence upon activation by MMP cleavage, demonstrating dose-dependent sensitivity to IL-1β-induced MMP activity in cultured human chondrocytes. In vivo studies using a destabilization of the medial meniscus (DMM) mouse model of OA highlighted the nanoparticle's efficacy. Fluorescence intensity in the OA knee joint significantly surpassed that in sham-operated knees, with a steady increase observed from 2 to 8 weeks post-surgery. By week 8, fluorescence intensity was approximately 3.5 times higher in OA knees compared to controls, confirming progressive MMP activation. In the requirements of post-traumatic OA (PTOA) diagnosis and treatment growing, Zhou et al. [117] developed an integrated diagnostic and therapeutic micelle system ERMs@siM13 for the early diagnosis and intervention of PTOA (Fig. 3C). The micelle structure includes a MMP13-cleavable polyethylene glycol shell, a black hole quencher-3, a cyanine 5 fluorophore, cyclic RGD ligands, and cationic lipids encapsulating MMP13-targeted small interfering RNA (siM13). The imaging principle leverages MMP13 activity in diseased cartilage, where MMP13 cleaves the PEG shell, restoring Cy5 fluorescence and exposing cRGD ligands. This enhances micelle binding to αvβ3 integrins on the chondrocyte surface and promotes cellular uptake. Experimental results demonstrated a significant reduction in MMP13 expression (to 23.66 % of the PBS group) and a marked increase in type II collagen (Col 2) levels to 4.06-fold (at 4 weeks) and 6.04-fold (at 8 weeks) compared to the PBS group (Fig. 3D). Biosafety evaluation showed no synovial inflammation, no histological abnormalities in major organs, and normal blood parameters following intraarticular administration, indicating good local and systemic biocompatibility.

### 4.3 Proteoglycans

Proteoglycans (PGs) are among the most crucial biomolecules in the cartilage matrix responsible for maintaining the structure and function of cartilage. PGs consist of a protein core and multiple glycosaminoglycan (GAG) side chains, including primarily chondroitin sulfate (CS) and keratan sulfate, with CS being the predominant GAG in cartilage. PGs contribute to cartilage's turgor and elastic properties by attracting water through their fixed charge density and cartilage's load-bearing capacity maintenance through electrostatic repulsion [118]. In healthy cartilage, the presence of proteoglycans maintains the elasticity and functionality of the tissue. However, the degradation of proteoglycans is among the earliest biochemical changes during the pathological progression of OA. Thus, the degradation of proteoglycans is a critical biomarker in the progression of OA.

**Fig. 4:**
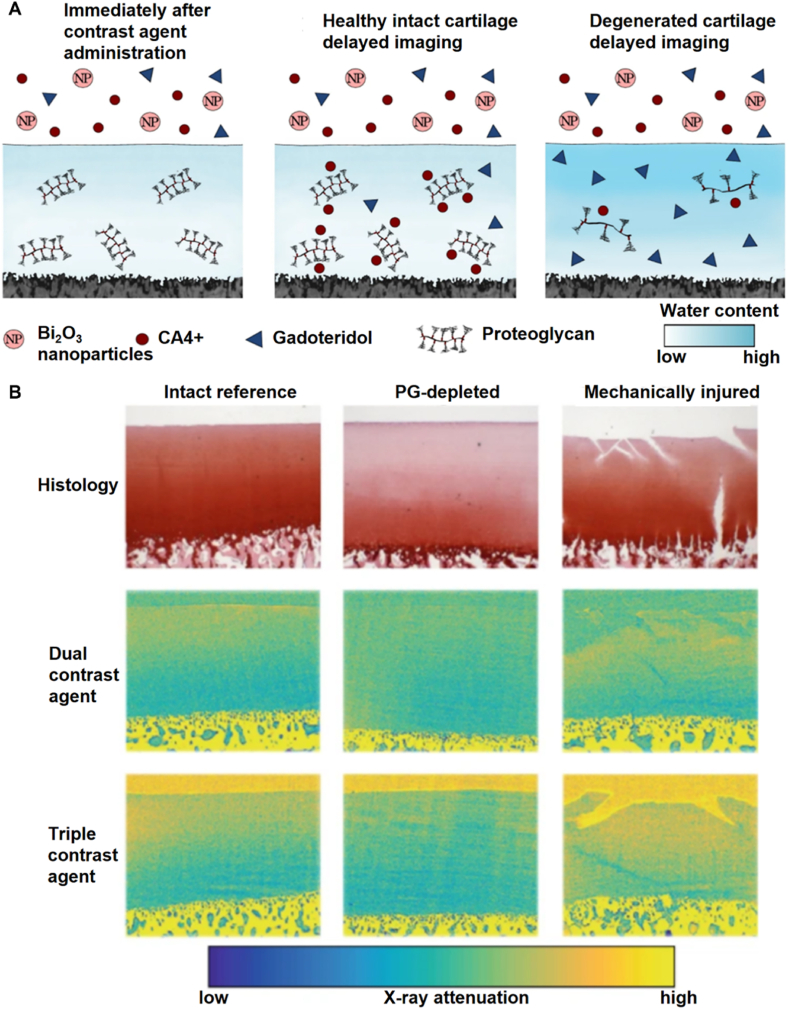
**Imaging nanoparticle targeting PG. A)** Mechanism of the combination of the three nanoparticles to enhance imaging performance [119]. **B)** The μCT (32 keV) images of the intact reference, PG-depleted, and mechanically injured cartilage after injecting dual contrast agent and triple contrast agent [119]. Adapted from Ref. [119] with permission.

Several works employ this strategy of using PG-recognizing nanoparticles as a contrast agent to improve the resolution and tissue contrast of OA imaging. For example, Honkanen et al. [119] introduced a triple-contrast computed tomography (CT) method leveraging a combination of cationic iodine-based CA^4+^, non-ionic gadoteridol, and bismuth nanoparticles (BiNPs) to enable simultaneous evaluation of proteoglycan (PG) and water contents in articular cartilage (Fig. 4A and B). Their method aims to overcome limitations of dual-contrast CT by providing enhanced segmentation of articulating surfaces and improving sensitivity to cartilage degeneration. Positively charged CA^4+^ targets the negatively charged PGs by the electrostatic force. Gadoteridol, a non-ionic gadolinium-based contrast agent, indicates water content as it distributes within the cartilage according to hydration and steric hindrance, allowing quantification via dual-energy CT (QDECT). BiNPs were then used to remain at the cartilage surface to enhance boundary contrast. The ex vivo bovine cartilage model demonstrated that the triple-contrast agent effectively quantified PG depletion and mechanical damage while maintaining segmentation accuracy. Notably, at the 2-h mark, the triple contrast agent improved cartilage imaging, making it easier to see surface details and cracks. The BiNPs maintained stability, with only a small, non-significant size increase from 193 ± 2 nm to 205 ± 4 nm after 24 h. CA^4+^ uptake was significantly higher in healthy cartilage, particularly in the superficial and middle zones. In contrast, gadoteridol exhibited increased uptake in the deep zones, especially in PG-depleted cartilage. Normalizing CA^4+^ with gadoteridol improved differentiation between healthy, PG-depleted, and injured cartilage. In terms of biosafety, the authors noted that although gadoteridol and CA^4+^ have shown favorable safety profiles individually, and bismuth exhibits low toxicity, the combined formulation still requires further evaluation.

In other work, Lin et al. [120] reported on anionic iodinated contrast agents, specifically ioxaglate which is commercially known as Hexabrix™, for equilibrium partitioning of ionic contrast agents via micro-computed tomography (EPIC-μCT). These agents, consisting of anion nanocarriers, repulse with the negatively charged PG to reduce the concentration of the contrast agent at the saturated PG area in cartilage, which allows for precise visualization of cartilage morphology and biochemical composition. The Hexabrix™ system enables quantitative evaluation of cartilage volume, thickness, and PG content by correlating X-ray attenuation with PG depletion in degraded areas. For instance, EPIC-μCT using Hexabrix™ generated high-resolution three-dimensional cartilage images, offering a non-destructive and quantitative alternative to traditional histopathology. Jäntti et al. [15] introduced cationic tantalum oxide nanoparticles (Ta_2_O_5_-cNPs) as μCT contrast agents for quantitative imaging of articular cartilage by specifically targeting PG distribution and collagen network organization. Its key mechanism relies on electrostatic attraction. Since PG carries a fixed negative charge, positively charged Ta_2_O_5_-cNPs can selectively aggregate in PG-rich areas. However, the collagen network also affects the diffusion of this contrast agent. The opener structure in the surface layer facilitates contrast agent penetration, while the denser collagen content part affects permeability and hinders diffusion. In addition, Ta_2_O_5_-cNPs exhibit strong X-ray attenuation at its k-edge (67.4 keV), which has higher contrast resolution than traditional CT agents. In the horse cartilage model, the maximum partition coefficient (Pmax) of Ta_2_O_5_-cNPs was closely related to the PG content (ρ = 0.87, p < 0.001) and equilibrium modulus (ρ = 0.80, p < 0.001). At the same time, its diffusion gradually slowed down with depth, and the diffusion rate at 50 % and 80 % depth was reduced by 2.6 times and 3.7 times relative to the surface, respectively. These findings indicate that Ta_2_O_5_-cNPs can perform non-destructive, depth-resolved 3D imaging of cartilage, providing new ideas for early detection of OA.

Briat et al. [121] also developed a radiotracer ^99m^Tc-NTP 15-5 as a contrast agent to sensitively investigate the changes in PG concentration level. The agent is the combination of a positively charged quaternary ammonium group and a polyazamacrocycle for technetium-99m labeling. The positive charge on the agent can target to the negatively charged PG through electrostatic attraction force. Thus, the agent can accumulate at the area in the cartilage where the concentration of PG is high and varies with the change of the level of PG. Many joint structures in mice (such as knees, humeral heads, and intervertebral discs) showed the accumulation of ^99m^Tc after 30mins intravenous injection of 20 MBq ^99m^Tc-NTP 15-5. This result demonstrates that the radiotracer provides early and sensitive assessment of cartilage remodelling.

### 4.4 Glycosaminoglycans

**Fig. 5:**
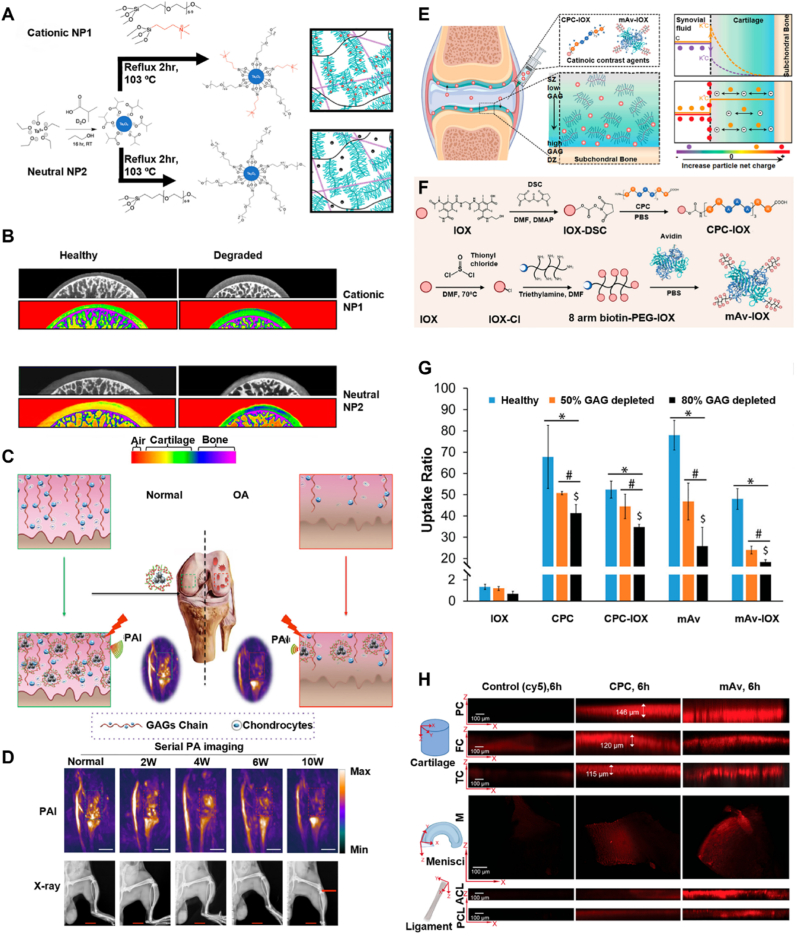
**Imaging nanoparticles targeting GAGs**. **A)** Synthesis progress of both cationic and neutral Ta_2_O_5_ nanoparticles [122]. **B)** Colour map and corresponding contrast-enhance CT slice of coronal slices of MCPJs of healthy cartilage and OA cartilage samples [122]. **C)** PLL-MNPs enhance PAI imaging mechanism [123]. **D)** PAI and X-ray imaging timeline [123]. **E)** Mechanism of CPC–IOX and mAv–IOX contrast agent penetrating the whole cartilage [81]. **F)** Synthesis scheme of CPC-IOX and mAv-IOX [81]. **G)** In vivo biodistribution of Cy5-labeled CPC, Texas-Red-labeled mAv, and free dye (Cy5 or Texas-Red) after injecting into healthy rat knee joint for 6h [81]. **H)** CPC and mAv distribution in joint area after injection for 6h [81]. Adapted from Refs. [81,122,123] with permission.

Nanoparticle-based contrast agents that target glycosaminoglycans (GAGs) can enhance resolution and tissue contrast in imaging degenerative conditions like OA. While GAGs are a major component in PGs, they also independently play an important role in water retention in the extracellular matrix (ECM). For instance, Lawson et al. [122] developed a novel nanoparticle-based contrast agent system using cationic and neutral tantalum oxide nanoparticles (Ta_2_O_5_ NPs) for advanced imaging and quantitative assessment of OA (Fig. 5A and B). The cationic nanoparticles (NP1), functionalized with tetra-alkyl ammonium ligands, target anionic glycosaminoglycans (GAGs) in cartilage via electrostatic interactions to achieve strong correlations between CT attenuation and GAG content (R^2^ = 0.89) as well as equilibrium modulus (R^2^ = 0.83). Neutral nanoparticles (NP2), designed to diffuse based on cartilage porosity, then complement NP1 by providing insights into structural changes. In ex vivo human cartilage models, NP1 achieved rapid and uniform diffusion across cartilage layers within 24 h, offering a dynamic attenuation range 1.33 times greater than NP2 at low concentrations. This molecule-targeting system overcomes limitations of plain radiography by simultaneous visualization of cartilage and subchondral bone, thus allowing for the detection of subtle cartilage degradation in early OA. Cytotoxicity was evaluated using NIH3T3 fibroblast cells, with 24-h exposure resulting in IC_50_ values of 24.29 mg/mL for NP1 and 20.07 mg/mL for NP2, indicating low acute toxicity and supporting their suitability for biomedical imaging applications.

In other work, Xiao et al. [123] designed melanin-based nanoparticles modified with cationic poly-l-Lysine (PLL-MNPs) (Fig. 5B). PLL-MNPs acting as cationic probes can selectively bind to these negatively charged GAGs, allowing their distribution and concentration in cartilage to be visualized by photoacoustic imaging (PAI). During the progression of OA, changes in GAGs content led to variation in photoacoustic signal intensity. This correlation enables PLL-MNPs-enhanced PAI to sensitively track the progression of OA by detecting changes in GAGs levels. PLL-MNPs displayed a remarkable sensitivity to GAG concentration changes, enabling in vitro and in vivo monitoring of cartilage degeneration at a low concentration of 0.5 mg/mL. In a destabilized medial meniscus mouse model injected with PLL-MNPs, PAI detected a consistent decrease in PA signal intensity corresponding to OA progression, which diagnose cartilage damage 4 weeks earlier than traditional X-ray imaging (Fig. 5C). Histological analysis confirmed that GAG depletion and cartilage damage have a strong correlation to PAI intensity. Toxicity and biosafety evaluations demonstrated that PLL-MNPs possess low cytotoxicity and good biocompatibility. Biodistribution studies revealed transient accumulation mainly in the liver and spleen with no abnormal retention in other major organs, and the nanoparticles were largely cleared from the body within 96 h. This nanoparticle-based imaging platform enables high specificity and sensitivity for real-time, non-invasive tracking of OA progression, holding a significant potential for theranostics and clinical translation in personalized medicine.

Furthermore, Zhang et al. [81] developed a cationic nanoparticle-based contrast agent system to effectively penetrate cartilage tissue through charge-driven mechanisms to enhance CT imaging for early OA diagnosis. It consists of cationic peptide carriers (CPC) and multi-arm PEGylated Avidin (mAv) nanoparticles, conjugated to the anionic contrast agent ioxaglate (IOX). Its net positive charge interacts with the negatively fixed charge density of cartilage, creating steep concentration gradients at the synovial fluid-cartilage interface via Donnan partitioning to drive efficient drug delivery (Fig. 5E and F). The weak-reversible electrostatic binding prevents excessive retention in cartilage surface, while the PEGylated structure enhances stability and biocompatibility. These nanoparticles achieve rapid full-thickness penetration into cartilage within 6 h, showing 20–50 times higher uptake at remarkably low iodine concentrations of 0.5–1 mg/mL (Fig. 5E). The requirement of IOX for imaging is 50–100 times lower than conventional anionic IOX which can greatly reduce toxicity risks. In rat tibial joint models, CPC–IOX nanoparticles demonstrated precise delineation of cartilage from subchondral bone and strong correlations between CT attenuation and GAG content, enabling high-resolution mapping of cartilage degradation (Fig. 5H). This innovative nanoparticle platform provides a highly sensitive and safer approach for early OA detection and disease progression monitoring.

### 4.5 Folate receptors and C-type lectin receptors on macrophages

Folate receptors (FRs) are a class of glycoproteins located on the cell surface, anchored by glycosylphosphatidylinositol (GPI), and mainly responsible for high-affinity binding to folic acid and its reduced cofactors. There are four types of folate receptors in humans: FR-α, FR-β, FR-γ, and FR-δ. Among them, FR-α, FR-β, and FR-δ are anchored to the cell membrane through GPI, while FR-γ mainly exists in a secreted form. It is worth noting that folate receptor β (FRβ) is a GPI-anchored plasma membrane protein that is selectively expressed on bone marrow cells. It has a high affinity for folate, making it an ideal candidate for targeted binding of folate-based PET tracers [124,125]. C-type lectin receptors are transmembrane proteins with at least one C-type lectin-like domain. They are primarily involved in recognizing microbial carbohydrates and promoting phagocytosis. Key members such as Dectin-1 and Dectin-2 play key roles in pathogen binding, immune signalling, and antigen presentation. They therefore play a vital role in innate immunity and host defence [126]. Macrophages are an important cell type in the immune system, responsible for engulfing pathogens, clearing damaged tissues, and regulating inflammatory responses and tissue repair [127]. In OA, polarized macrophages show high expression of folate receptors, which can be targeted and imaged in a specific manner for precise diagnosis and monitoring [128,129]. Thus, folate receptors show important potential value as a biomolecule target in macrophage imaging.

**Fig. 6:**
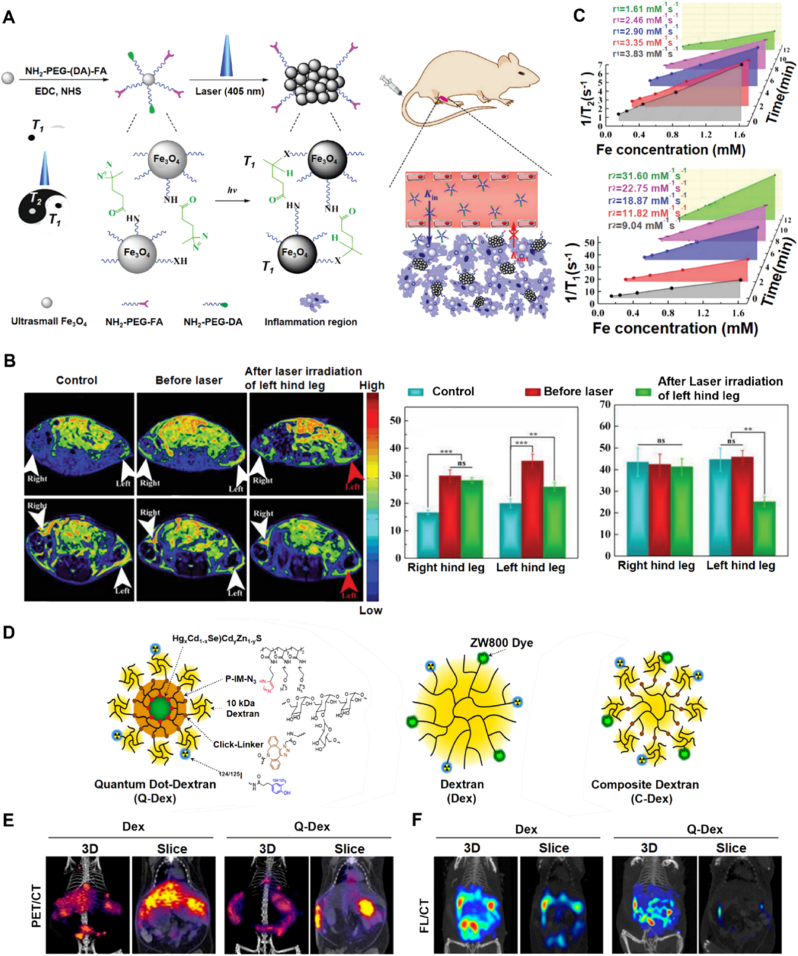
**Imaging nanoparticles targeting folate receptor and C-type lectin receptors on macrophages. A)** Schematic graph of the synthesis of Fe_3_O_4_-PEG-(DA)-FA NPs for enhancing T_1_/T_2_-weighted MR imaging of inflammatory arthritis [16]. **B)** T_1_/T_2_-weighted MR images of the arthritis model [16]. **C)** T_1_ and T_2_ relaxion rate [16]. **D)** Illustration of chemical structure of dextran-mimetic QD probes (Q-Dex) and all-organic dextran probe analogues (Dex and C-Dex) [130]. **E)** 24-h post-injection 3D PET CT images of obese mice after intraperitoneal injection of 10 nmol Dex or Q-Dex with cross-sectional views [130]. **F)** 3D FL images overlaid on CT scans of the same mice focusing on the peritoneal cavity IVIS Adapted from Refs. [16,130] with permission.

Li et al. [16] designed photoresponsive ultrasmall iron oxide nanoparticles (Fe_3_O_4_-PEG-(DA)-FA NPs) to improve T_1_/T_2_-weighted MRI imaging resolution in inflammatory arthritis, with enhanced T_1_ signals observed in the inflamed joint after laser irradiation, indicating successful activation of the contrast agent and improved imaging sensitivity at the target site. (Fig. 6A and B). These Fe_3_O_4_-PEG-(DA)-FA NPs can form nanoclusters (NCs) and accumulate in inflamed joints under 405 nm laser irradiation. The nanoparticles specifically targeted macrophages through FR-mediated endocytosis, utilizing FR as a biomarker highly expressed in activated macrophages associated with inflammatory arthritis. The experimental results showed that after 12 min of laser irradiation, the formation of NCs led to a decrease in the r_1_ relaxation rate from 3.83 to 1.61 mM^−1^s^−1^ and a significant increase in the r_2_ relaxation rate from 9.04 to 31.6 mM^−1^s^−1^ (Fig. 6 C). Experimental results of targeted T_1_-weighted MRI imaging of inflammatory arthritis showed that the nanoparticles had stronger enrichment and longer retention time at the arthritis site compared with the free folic acid (FA) pre-blocked model. This property enabled the MRI signal to remain enhanced within 30–60 min and still be detectable at 120 min. Quantitative analysis showed that the MRI signal-to-noise ratio (SNR) of the common arthritis model increased by 1.79–2.66 times, indicating that the nanoparticles can significantly enhance T_1_-weighted MRI imaging (Fig. 6 C). This folate receptor-targeted contrast agent significantly enhanced the retention of nanoparticles in arthritic areas and improved the sensitivity and specificity of MRI. In vitro cytotoxicity assays demonstrated that Fe_3_O_4_-PEG-(DA)-FA NPs maintained over 86 % cell viability at Fe concentrations up to 3.0 mM, indicating excellent cytocompatibility. Moreover, no significant inflammatory response or increase in glycosaminoglycan (GAG) loss was observed during an 8-day cartilage culture, supporting the biosafety of this nanoplatform.

Another strategy uses Q-Dex quantum dot (QD) probes which employ “click chemistry” to specifically target and combine radioactive iodine for multimodal imaging capabilities. Deng et al. [130] developed dextran-mimetic quantum dots (Q-Dex), composed of a near-infrared-emitting crystalline core-shell structure [(HgxCd1–xSe)CdyZn1–yS] coated with a multidentate azide-functional polymer (P-IM-N3). This kind of polymer can conjugate to dextran via strain-promoted click chemistry (Fig. 6D). The nanoparticle exhibited the ability to target macrophages through an endocytosis mechanism mediated by the interaction between C-type lectin receptors, particularly DC-SIGN and L-SIGN, expressed on macrophages and the dextran coating on the nanoparticle surface. Compared to native dextran, Q-Dex exhibited a 9-fold increase in blood circulation half-life, superior photostability (no significant photobleaching after 98 cycles of imaging), and improved fluorescence quantification accuracy in tissues (2.3-fold better agreement with gold standard gamma well counting). Additionally, Q-Dex achieved higher signal-to-noise ratios and maintained fluorescence in chemically fixed tissues, enabling high-resolution 3D imaging of macrophages in inflamed visceral adipose tissue (Fig. 6E and F). This demonstrates the great potential of Q-Dex for detecting inflammatory diseases. Importantly, Q-Dex showed no acute cytotoxicity in macrophages and adipocytes at imaging-relevant concentrations (≤50 nM), and its protective dextran/polymer shell effectively prevented heavy metal ion leakage, demonstrating excellent biocompatibility.

## 5 AI in medical imaging and nanomedicine

### 5.1 The role of AI in medical imaging

AI, especially deep learning technology, has demonstrated significant potential in OA imaging by automating complex image analysis tasks [[12], [13], [14]]. Recently, Heppenstall et al. [131] conducted a large-scale study using DXA scans from 40,312 participants in the UK Biobank, where they applied a deep learning algorithm to automatically extract geometric parameters of the hip joint, such as femoral neck width, femoral head diameter, and hip axis length. These parameters were further analyzed using logistic regression and Cox proportional hazards models, revealing significant associations with radiographic hip OA, hospital-diagnosed hip OA, and total hip replacement, independent of body size and other confounding factors. Similarly, AI techniques are leveraged to analyze knee X-ray data for KL grading, joint space width measurement, and osteophyte detection, significantly reducing image reading time. Nehrer et al. [132] evaluated a deep learning-based computer-aided diagnosis system (KOALA, developed by IB Lab GmbH) that utilizes convolutional neural networks to automatically assess radiographic features relevant to OA, including KL grade and OARSI scores for joint space narrowing, sclerosis, and osteophytes. In a controlled reader study using 124 radiographs from the OA Initiative (OAI), three experienced physicians scored the images both unaided and aided by KOALA. The results demonstrated that AI assistance improved inter-reader agreement for KL grading from an intraclass correlation coefficient (ICC) of 0.67–0.81, and enhanced diagnostic accuracy for KL > 1 from 0.76 to 0.82, primarily by improving specificity from 0.65 to 0.88 without compromising sensitivity. This suggests that CNN-based tools can standardize KL grading and reduce interobserver variability in radiographic OA diagnosis. Elsewhere, Olsson et al. [133] trained a deep convolutional neural network using a ResNet architecture on 6103 knee radiographs collected from routine clinical imaging at a Swedish hospital, including cases with common visual disturbances such as implants or casts. The model was evaluated on an independent test set of 300 images labeled by two senior orthopedic surgeons. The AI model achieved a mean AUC of 0.92 across all KL grades and AUCs above 0.95 when adjacent KL grades were merged. These results, derived from a real-world, unfiltered dataset, indicate that CNN-based models can achieve expert-level classification performance in knee OA, even in heterogeneous clinical imaging conditions.

In another study, Pedoia et al. [134] trained a densely connected convolutional neural network (DenseNet) to classify radiographic knee OA using T_2_ relaxation time maps from 4384 subjects in the OA Initiative (OAI) baseline cohort. The deep learning model was trained on fully automated, flattened T_2_ maps of four cartilage compartments and integrated demographic features (age, gender, BMI, KOOS pain score). On an independent test set, the model achieved an AUC of 83.44 %, with sensitivity of 76.99 % and specificity of 77.94 %. This significantly outperformed the best shallow classifier using PCA-derived features and random forests (AUC = 77.77 %, sensitivity = 67.01 %, specificity = 71.79 %), as confirmed by McNemar’s test (P = 0.0013). These findings demonstrate that deep learning applied to quantitative MRI can extract spatial relaxometry patterns beyond conventional average-based methods, providing a promising framework for automated OA diagnosis using compositional cartilage imaging.Furthermore, Almajalid et al. [135] developed a fully automatic deep learning system for detecting and segmenting knee bones from three-dimensional magnetic resonance imaging (MRI) sequences using a modified U-net architecture. The model was trained and tested on 99 knee DESS-MRI cases (15,840 slices) from the OA Initiative (OAI) dataset, encompassing tibia, femur, and patella bone structures. A two-stage approach was implemented: first, a detection network identified relevant bone slices across 160-slice volumes, followed by a segmentation network that extracted bone contours. On the independent test set, the method achieved a slice-level detection accuracy of 98.79 %, and a segmentation performance of 96.94 % Dice coefficient and 93.98 % similarity index for full-knee bone segmentation. Compared to other state-of-the-art models including original U-net, SegNet, and FCN-8, the proposed model showed significantly higher accuracy (p < 0.00001). These results demonstrate the feasibility of fully automated bone segmentation from 3D knee MRI and establish a foundation for subsequent cartilage and biomarker analysis in OA research. There are more studies suggest that AI can significantly improve the processing efficiency when facing a large-scale imaging dataset and enhance the diagnostic accuracy and consistency of OA [136,137].

Furthermore, the integration of multimodal imaging technology with AI provides potential for comprehensive evaluation of joint structure and function. For example, EPIC-μCT technology can measure the distribution of chondroproteoglycans through ionic contrast agents, and AI and deep learning algorithms can integrate this data to generate quantitative assessments. This approach enables a more holistic understanding of osteochondral structural and biochemical changes [52,138]. In addition, researchers have developed matrix metalloproteinase (MMPs) targeted fluorescent probes combined with AI technology for image analysis, which could facilitate real-time monitoring of key enzyme activities, thereby providing more valuable information in early diagnosis and treatment effect evaluation [139,140].

### 5.2 The role of AI in nanoparticle design

AI is transforming nanomedicine by streamlining the design, optimization, and evaluation of nanoparticle-based drug delivery systems [141]. Traditional nanoparticle research relies on trial and error, which is time-consuming and inefficient [142]. AI-driven models can not only accelerate design, predict optimal nanoparticle structures, but also optimize material selection through large-scale data analysis. Beyond design, AI also enhances biological interaction analysis by modeling protein corona formation and cellular uptake, enabling more accurate predictions of biodistribution [143]. In addition, AI improves therapeutic effect prediction, evaluating and analysing drug release kinetics, nanoparticle accumulation in target tissues, and toxicity of nanoparticles [144]. Finally, AI-powered high-throughput screening and database integration can optimize formulation selection, reduce experimental workload, and improve experimental precision.

For example, Moore et al. [145] explored the integration of AI and mathematical modelling to optimize the design of gold nanoparticles (AuNPs) in medical biophysics, particularly in cancer diagnosis and treatment. Machine learning (ML) algorithms, including Monte Carlo simulations, were used to simulate the aggregation, diffusion, and receptor binding of nanoparticles in biological environments. AI-driven models simulated the aggregation, diffusion, and receptor binding of nanoparticles in biological environments, thereby optimizing their therapeutic effects. Bayesian neural networks and deep learning frameworks were used to improve nanoparticle surface modifications, enhancing drug encapsulation efficiency, cellular uptake, and minimize cytotoxicity. SPR modelling determined that AuNPs exhibited peak absorption at 520 nm, ensuring excellent light absorption in photothermal therapy. AI-assisted mathematical models were used to optimize nanoparticle surface modifications, enhance targeted drug delivery, and improve gene therapy efficiency. Experimental results showed that plasmid DNA (pDNA) uptake efficiency peaked at an AuNP/DNA ratio of 2400:1, while cellular uptake analysis indicated that 50 nm Au NPs exhibited higher internalization efficiency, with 6 × 10^3^ nanoparticles per cell. These AI-driven approaches facilitate nanoparticle optimization, minimize experimental iterations, and promote advances in nanomedicine research.

In addition, Vilanova et al. [143] integrated experiments, computer simulations and theoretical analysis to study the formation dynamics and composition of the protein-nanoparticle corona. Based on the principle of protein competitive adsorption, the research team used silica nanoparticles and three plasma proteins (human serum albumin HSA, transferrin Tf, and fibrinogen Fib) to conduct experiments, and used differential centrifugal sedimentation (DCS) and microscale thermophoresis (MST) to determine the binding affinity of proteins. At the same time, the study predicted the long-term evolution of the protein corona by coarse-grained molecular simulation (CG) and non-Langmuir differential rate equation (NLDRE). The experimental results not only show that the adsorption kinetics of Fib is affected by the concentration of competing proteins but also reveal that the final composition of the protein corona is affected by the early protein adsorption order (memory effect). This study shows that theoretical modeling combined with computer simulation (CG + NLDRE) can be used to predict protein adsorption patterns and improve the design efficiency of nanoparticles in biomedical applications (such as targeted drug delivery and personalized nanomedicine). In terms of drug penetration and delivery mechanisms, Zhu et al. [146] used an image segmentation-based method (nano-ISML) to perform machine learning-assisted analysis on 67,530 blood vessels in 32 tumour models and found that vascular permeability was highly heterogeneous. Their results indicated that tumours with greater vascular permeability exhibited significantly improved nanoparticle permeability. The ratio of high-permeability blood vessels among different tumours varied by more than 13 times, while the nanoparticle permeability between the highest and lowest permeability blood vessels differed by over 100 times. The machine learning model achieved an accuracy of about 90 % in blood vessel segmentation and about 80 % in nanoparticle penetration analysis, and was able to divide blood vessels into high permeability (VP > 0.6), medium permeability (0.3 ≤VP ≤ 0.6), and low permeability (VP < 0.3), while tumours were divided into high permeability (PR ≥ 4) and low permeability (PR < 4). This study highlights how machine learning can facilitate high-throughput quantitative analysis, providing insights into the rational design of personalized drug delivery strategies.

In another study, Ahmadi et al. [147] leveraged machine learning to predict the toxicity of nanoparticles, to reduce the cost and time consumption of experimental methods. The research team built a data set based on the NanoHub database, which contained 244 records covering a variety of nanoparticle types, physicochemical properties, exposure conditions and cellular responses. They applied five machine learning models: decision tree (DT), random forest (RF), support vector machine (SVM), naive Bayes (NB) and artificial neural network (ANN), and used the Gini index to evaluate the impact of each factor on toxicity. The results showed that cell line, exposure dose and tissue type were the key factors determining cytotoxicity, and the RF model had the best prediction performance, with an accuracy of 93.45 % and an AUC value of 0.966. This study demonstrated the potential of machine learning in nanotoxicity assessment.

Although the integration of AI and nanomedicine has demonstrated remarkable potential in optimizing drug delivery and formulation, several challenges remain. These include the need for standardized nanoparticle characterization methods, the difficulties of integrating multi-source biological and experimental data, and cost constraints that could hinder the clinical translation of these technologies [148,149]. AI for nanomedicine design specially designed for OA has seldom been reported, presenting a potential new direction for the AI application in this field.

### 5.3 AI limitations and ethical concerns

Although AI has shown significant potential in OA image analysis and multimodal data processing, it still faces many limitations and ethical challenges in practical applications. First, the training of AI models is highly dependent on large-scale, high-quality datasets, which often come from specific populations or regions, resulting in underrepresentation of the models. When these models are applied to groups of different races, ages, or disease subtypes, their performance may be significantly reduced, thereby amplifying existing medical inequalities [133,150]. In addition, most deep learning models are "black box models" and lack clear interpretability. This makes it possible for clinicians to face trust issues when using AI to make critical decisions (such as surgical recommendations or early OA screening). Ou et al. [150] emphasized that the opaque nature of AI decision-making significantly limits its clinical applicability, highlighting the urgent need for enhanced interpretability to ensure trustworthy clinical integration.

In terms of data security, the use of large amounts of clinical imaging and omics data raises privacy concerns. Even without direct data access, AI models may indirectly infer sensitive personal information from shared model parameters or gradients. Federated learning (FL) addresses some privacy concerns by allowing models to be trained across multiple institutions without data sharing. However, FL also faces challenges, such as susceptibility to model inversion attacks and difficulty in ensuring performance reliability due to heterogeneous datasets across institutions [151]. Rehman et al. also highlighted that FL has difficulty handling non-independent and identically distributed (non-IID) data, which reduces its accuracy and limits its promotion and application in clinical practice [152].

Another major limitation is the inadequate integration of multimodal data sources (clinical records, imaging, omics) by existing AI models. Current models typically only handle single-modal tasks, but OA is a complex degenerative disease determined by multiple factors, and single-modal models are unlikely to have a comprehensive understanding of OA. Ou et al. pointed out this limitation and pointed out that multi-task learning frameworks and deeper integration of different data sources are urgently needed [150]. Furthermore, traditional statistical methods in omics studies often assume linear interactions, which is insufficient to capture the complex nonlinear biological mechanisms of OA. This deficiency highlights the need for more sophisticated, AI-driven analytical approaches [150].

Ethical considerations must also be carefully addressed. Dataset biases may lead to discriminatory AI models, which could negatively impact healthcare equality. Furthermore, ethical debates regarding accountability for AI-assisted clinical decision making remain unresolved, which could lead to legal and liability challenges [150,153]. Therefore, developing clear ethical guidelines and regulatory frameworks is critical to ensuring transparency, fairness, and accountability of AI. Ou et al. advocated that interpretability, privacy, and fairness issues should be systematically addressed in future AI development to ensure responsible and trustworthy clinical deployment [150]. Rehman et al. also suggested incorporating strong governance and traceability mechanisms into federated learning systems to increase transparency and build clinical trust [152].

In summary, future developments in AI for OA research should address dataset heterogeneity, enhance model interpretability and privacy protection, promote multimodal data integration, and develop strong ethical guidelines. Addressing these challenges is critical to realizing the full potential of AI in OA diagnosis, treatment, and management [153].

## 6 Conclusions and perspectives

This review provides an overview of the pathology and pathophysiology of OA, the vital role of imaging technologies in OA evaluation, and how advanced nanoparticle designs can overcome current imaging limitations. The integration of imaging methods, selective biomarkers, and nanoparticle technologies offers promising approaches for early diagnosis and precise treatment of OA and potentially other diseases. A variety of molecular markers related to the course of OA (such as proteoglycans, glycosaminoglycans, type II collagen and inflammatory factors) have been identified as important targets for imaging technology. Many different targeting molecules are combined with various functional nanoparticles to create imaging agents that can accurately accumulate in the lesion site, thereby significantly improving the sensitivity and specificity of diagnosis.

Although significant progress has been achieved in recent years in integrating biomaterials with molecular biomarkers, many of the imaging technologies mentioned in this article remain in the early stages of development and have yet to transitioned into the clinical stage. Overcoming biological barriers, improving the biosafety and functionality of nanoparticles, establishing imaging standardization and long-term clinical verification are still urgent issues to be solved in this field. In addition, the application of AI can help analyze and integrate multimodal imaging data to improve diagnostic efficiency.

### 6.1 Targeting ability

The functionalization of nanoparticles usually relies on physical adsorption or covalent coupling technology to achieve the targeting ability. In physical adsorption, functional ligands are anchored on the surface of nanoparticles via nonspecific adsorption. This method may lead to uneven distribution of ligands, off-target detachment, and poor control over their orientation and density, which in turn affect targeting efficiency and imaging sensitivity. Covalent coupling firmly conjugates ligands to the surface of nanoparticles through chemical bonds, providing a more stable binding mode. However, this method has challenges in precise control of ligand conjugation i.e., antibody orientation. Also, covalent conjugation may affect the active part of the functional molecules, thereby hampering the targeting effect. To optimize the targeting ability, future research needs to focus on developing more precise surface modification technologies to achieve controllable ligand distribution and retain its biological activity. This will help further enhance the diagnostic and therapeutic potential of targeted nanomaterials in OA. Targeting specificity is another challenge. The choice of targeting ligand is critical to ensure the imaging probe specifically targets the selected biomarker which is often expressed in limited concentrations or in a heterogeneous manner across different sections of the same tissue or different stages of the disease.

### 6.2 Biocompatibility challenges

The biocompatibility of nanomaterials is one of the key challenges for clinical translation, since nanomaterials may induce cytotoxicity or immune response due to their chemical composition or surface properties. Therefore, optimizing surface modification technology can lead to improved biocompatibility and stability. However, even for modified nanomaterials, the long-term safety of their degradation byproducts in vivo still needs to be further verified. To address these issues, future research should focus on developing new nanomaterials with higher biocompatibility, such as biomimetic cell membrane-coated nanoparticles or fully biodegradable inorganic nanomaterials. These strategies can not only reduce toxic reactions but also enhance the adaptability of materials in joint tissues, thereby promoting their widespread application in the field of imaging.

### 6.3 Targeting beyond cartilage

The current research in molecular biomarker-targeting nanoparticle imaging for OA has largely focused on cartilage, given that the cartilage degeneration is a hallmark of OA. However, OA is a complex musculoskeletal disorder that affects the entire joint. Future research efforts should be invested on the other key joint structures, such as subchondral bone, synovium, ligaments, and periarticular tissues. One of the challenges hindering the scope beyond cartilage could be the limited penetration of nanoparticles into deeper joint tissues. Improving nanoparticle penetration and retention in deeper joint sites by tuning nanoparticle size, shape and surface chemistry could facilitate deep tissue penetration and improve access to the subchondral bone and other joint structures, which leads towards realizing the effective molecular imaging of the whole joint structure in OA.

### 6.4 Clinical translation

In addition to biosafety concerns, the development of nanoparticle–based imaging agents for clinical use faces significant challenges according to good manufacturing practice (GMP) compliance, scalability, and regulatory approval. Producing nanomaterials under GMP standards is essential to ensure batch-to-batch consistency, reproducibility, and quality control, which are critical factors for regulatory acceptance and clinical application. However, the complex preparation process of nanoparticles may lead to inconsistent yields and unstable physicochemical properties, compromising their imaging quality and safety. According to the FDA, nanomaterial-containing drug products must comply with GMP regulations, as specified in Title 21 of the Code of Federal Regulations (CFR), which outline the minimum requirements for the manufacturing, processing, packing, and holding of drug products. Manufacturers are required to establish well-defined critical quality attributes (CQAs) and in-process controls to ensure consistent quality, identity, strength, and purity of each batch. Changes in the size, morphology, or coating of nanoparticles during production scale-up could be considered as batch inconsistencies or even impurities and should be monitored through validated analytical techniques [154].

Recent achievements in microfluidic manufacturing technology have shown significant advantages in the scalable and controlled production of lipid nanoparticles, providing a potential pathway toward the large-scale fabrication of inorganic nanomaterials. Nevertheless, the translation of this technology to more structurally complex nanomaterials still require further optimization. The FDA also notes that nanomaterials are often sensitive to manufacturing scale and process conditions, making it essential to build risk-based control strategies early in development to support scale-up and prevent loss of function or safety. Retention of representative samples from early batches is recommended to establish comparability across development and commercial production. In parallel, integration of optimized material design and biocompatible scalable manufacturing processes could accelerate nanoparticle-based imaging agents' clinical performance and provide breakthrough technical support for the functional as well as precise diagnosis and early intervention of OA. Finally, clinical translation considerations are the last step to consider before successfully employing these imaging agents in real world clinical environments. FDA advises developers to engage in early regulatory consultation (e.g., through pre-Investigational New Drug (pre-IND) meetings or controlled correspondence) and to align product development with appropriate submission pathways, such as the 505(b)(2) New Drug Application pathway (used when relying partially on existing data) or the 505(j) Abbreviated New Drug Application pathway (used for generics), depending on product classification. Before this, extensive research needs to be undertaken to corroborate and confirm the results of successful nanoparticle imaging, which may require unique verification methodology. For products incorporating nanomaterials, demonstrating comparable bioavailability to an existing agent may not be sufficient; additional evidence, such as in vivo pharmacokinetic/pharmacodynamic studies or safety assessments of nanocarriers, may be required to satisfy regulatory standards, particularly under these pathways. These may be more broader issues, such as patient acceptance, general ease of use, and practical application protocols. Some of these issues can be mitigated in combination with above, such as patients may accept the treatment once guaranteed it is non-toxic and biocompatible. The easiest way to practical application will be to integrate within existing imaging preparation processes, which may involve longevity and circulation time requirements when designing the imaging nanoparticle.

In summary, the combination of biomarkers and imaging nanoparticles provides a critical opportunity for personalized diagnosis and treatment of OA. While solving the bottlenecks of existing technologies, exploring innovative applications in molecular diagnosis imaging of OA is expected to promote the development of precision medicine for OA, ultimately improving the life quality and life expectancy of patients with OA.

## CRediT authorship contribution statement

**Tianrui Zhang:** Writing – review & editing, Writing – original draft, Visualization, Validation, Resources, Methodology, Investigation. **Qianyi Zhang:** Writing – review & editing, Writing – original draft, Visualization, Validation, Methodology, Investigation, Conceptualization. **Jingqian Wei:** Writing – review & editing, Writing – original draft, Visualization, Investigation. **Quanbin Dai:** Writing – review & editing, Supervision, Investigation. **Dzenita Muratovic:** Writing – review & editing, Validation, Investigation. **Wenjie Zhang:** Writing – review & editing, Validation, Resources. **Ashish Diwan:** Writing – review & editing, Supervision, Resources. **Zi Gu:** Writing – review & editing, Validation, Supervision, Resources, Project administration, Methodology, Investigation, Conceptualization.

## Declaration of competing interest

The authors declare that they have no known competing financial interests or personal relationships that could have appeared to influence the work reported in this paper.

## Data Availability

No data was used for the research described in the article.
